# The Potential Cardiometabolic Effects of Long-Chain ω-3 Polyunsaturated Fatty Acids: Recent Updates and Controversies

**DOI:** 10.1016/j.advnut.2023.03.014

**Published:** 2023-04-07

**Authors:** Jae Hyun Bae, Hyunjung Lim, Soo Lim

**Affiliations:** 1 Department of Internal Medicine, Korea University Anam Hospital, Korea University College of Medicine, Seoul, Republic of Korea; 2 Department of Medical Nutrition, Research Institute of Medical Nutrition, Graduate School of East-West Medical Science, Kyung Hee University, Yongin, Republic of Korea; 3 Department of Internal Medicine, Seoul National University Bundang Hospital, Seoul National University College of Medicine, Seongnam, Republic of Korea

**Keywords:** ω-3 FAs, EPA, docosahexaenoic acid, cardiometabolic risk factors, cardiovascular disease, atherosclerosis, metabolic dysfunction-associated fatty liver disease, gut microbiota

## Abstract

Various health-related effects of long-chain (LC) ω-3 PUFAs, EPA, and DHA have been suggested. LC ω-3 PUFAs reduce TG concentrations and have anti-inflammatory, immunomodulatory, antiplatelet, and vascular protective effects. Controversially, they might help in restoring glucose homeostasis via the gut microbiota. However, previous studies have not shown the clear benefits of LC ω-3 PUFAs for CVDs. REDUCE-IT and STRENGTH—representative randomized controlled trials (RCTs) that examined whether LC ω-3 PUFAs would prevent major adverse cardiovascular (CV) events (MACE)—showed conflicting results with differences in the types, doses, or comparators of LC ω-3 PUFAs and study populations. Therefore, we performed a meta-analysis using major RCTs to address this inconsistency and assess the clinical and biological effects of LC ω-3 PUFAs. We included RCTs that involved ≥500 participants with ≥1 y follow-up. Of 17 studies involving 143,410 people, LC ω-3 PUFA supplementation showed beneficial effects on CV death (RR: 0.94; 95% CI: 0.88, 0.99; *P* = 0.029) and fatal or nonfatal MI (RR: 0.83; 95% CI: 0.72, 0.95; *P* = 0.010). RCTs on EPA alone showed better results for 3-point MACE, CV death, and fatal or nonfatal MI. However, the benefits were not found for fatal or nonfatal stroke, all-cause mortality, and hospitalization for heart failure. Of note, studies of both the EPA/DHA combination and EPA alone showed a significant increase in risk of new-onset atrial fibrillation. Thus, well-designed studies are needed to investigate the underlying mechanisms involved in the distinct effects of EPA compared with DHA on cardiometabolic diseases. This review discusses the potential benefits and safety of LC ω-3 PUFAs from a cardiometabolic perspective focusing on recent updates and controversies.

**Statement of Significance**

This review provides a comprehensive discussion and practical considerations of the potential benefits and safety of ω-3 FAs from a cardiometabolic perspective focusing on recent updates and controversies. To reduce cardiovascular events, using EPA alone or combining EPA and DHA for therapeutic regimens are considered.

## Introduction

FAs are the major components of fats in human body. The physical and chemical characteristics and biological effects of FAs are greatly influenced by their types and proportions [1]. Several epidemiological and clinical studies have evaluated the relationship between EPA and DHA, 2 major compounds of long-chain (LC) ω-3 PUFAs, and cardiometabolic diseases, including hypertension, dyslipidemia, atherosclerosis, MI, heart failure, and arrhythmia [[2], [3], [4]]. However, previous studies have not shown the clear benefits of LC ω-3 PUFAs for CVD. Recently, 2 large clinical trials, the Reduction of Cardiovascular Events with Icosapent Ethyl-Intervention Trial (REDUCE-IT) [5] and the Long-Term Outcome Study to Assess Statin Residual Risk Reduction with EpaNova in High Cardiovascular Risk Patients with Hypertriglyceridemia (STRENGTH) [6], have also provided discrepant results. In REDUCE-IT, 4 g of icosapent ethyl (IPE), a highly purified form of EPA, produced cardiovascular (CV) benefits in people receiving statin therapy [5]. However, in STRENGTH, a high-dose combination of EPA and DHA did not show such benefits in people with high CV risk [6]. The conflicting results could be attributed to differences in the types (EPA plus DHA or EPA alone), doses, or comparators (corn oil, mineral oil, or other substances) of LC ω-3 PUFAs and distinct study populations.

Therefore, we reviewed the benefits and safety of the 20 to 22–carbon LC ω-3 PUFAs, EPA, and DHA from a cardiometabolic perspective focusing on recent updates and controversies, including those regarding atrial fibrillation. In addition, we conducted a meta-analysis using major randomized controlled trials (RCTs) of LC ω-3 PUFAs based on strict eligibility criteria to assess potential clinical implications. The results were also used to explain inconsistent evidence from RCTs and the clinical and biological effects of LC ω-3 PUFAs. Finally, we suggest a practical consideration in LC ω-3 PUFA therapy for cardiometabolic diseases.

## Characteristics of ω-3 PUFAs

### Synthesis of ω-3 PUFAs that compete with ω-6 PUFAs

FAs are classified as saturated or unsaturated. Saturated FAs have no double bonds, and each carbon forming the hydrocarbon chain has 2 hydrogen atoms and 2 adjacent carbon atoms. Unsaturated FAs have ≥1 double bond and are subdivided into monounsaturated FAs with one carbon-carbon double bond and PUFAs with 2 or more double bonds. Even if the carbon-carbon double bond is newly formed in the body, interconversion between the PUFA series, such as ω-3, ω-6, and ω-9 PUFAs, does not occur because it is created between the existing double bond and the carboxyl group [1].

ω-6 PUFAs may affect the efficacy of ω-3 PUFAs in humans. During the processing of ω-6 and ω-3 PUFAs, LA and ALA are converted into arachidonic acid and EPA by elongation and desaturation. Because both pathways use the same enzyme (Δ6-desaturase), competition exists between the 2 PUFA series to occupy the second position of phospholipids of the cells in nearly all tissues in humans in terms of conversion and storage [7]. It has been reported that the higher the ratio of LA to ALA, the more inhibited the synthesis of EPA and DHA from ALA [7]. In addition, arachidonic acid and EPA compete for the same enzymes in the conversion to active eicosanoids, some of which respond differently to ω-3 and ω-6 [7,8]. The process that exacerbates conditions leading to pathophysiology is more intense in the ω-6 motif than in the ω-3 motif [7]. For example, cyclooxygenase 1, an enzyme involved in PG formation, has a greater preference for ω-6 than ω-3 substrates and promotes PG_2_ and thromboxane A production, inducing an inflammatory response [7]. Because these are essential FAs, both the concentration of ω-3 and ω-6 PUFAs and their relative amounts make a difference in competition for conversion and storage. ω-3 and ω-6 PUFAs account for nearly 100% by weight of the composition of highly unsaturated FAs in blood or body tissues [9,10]. The range of ω-3 or ω-6 PUFAs is 15% to 85% in humans [9]. This relative amount plays an important role in inducing the competition between 2 FAs for conversion and storage [7]. The metabolism and role of these PUFAs are well described in a recent article by Lands [7].

In general, linoleic acid is sufficient in human diets but ALA is relatively lacking. Therefore, plasma and cell concentrations of LC ω-6 PUFAs tend to be higher than those of LC ω-3 PUFAs [8]. Based on limited studies, increasing the intake of ALA and decreasing the intake of LA in the diet have been suggested to improve LC ω-3 PUFA status [11]. Moreover, it may be helpful to reduce LA intake to <2.5% of energy to increase DHA concentrations [11]. However, although the relationship between ω-6 and ω-3 PUFAs might be important in human health, an agreed global standard suggesting an optimal ratio of ω-6 to ω-3 PUFAs for human health has not yet been determined [8].

### Sources of LC ω-3 PUFAs

**FIGURE 1:**
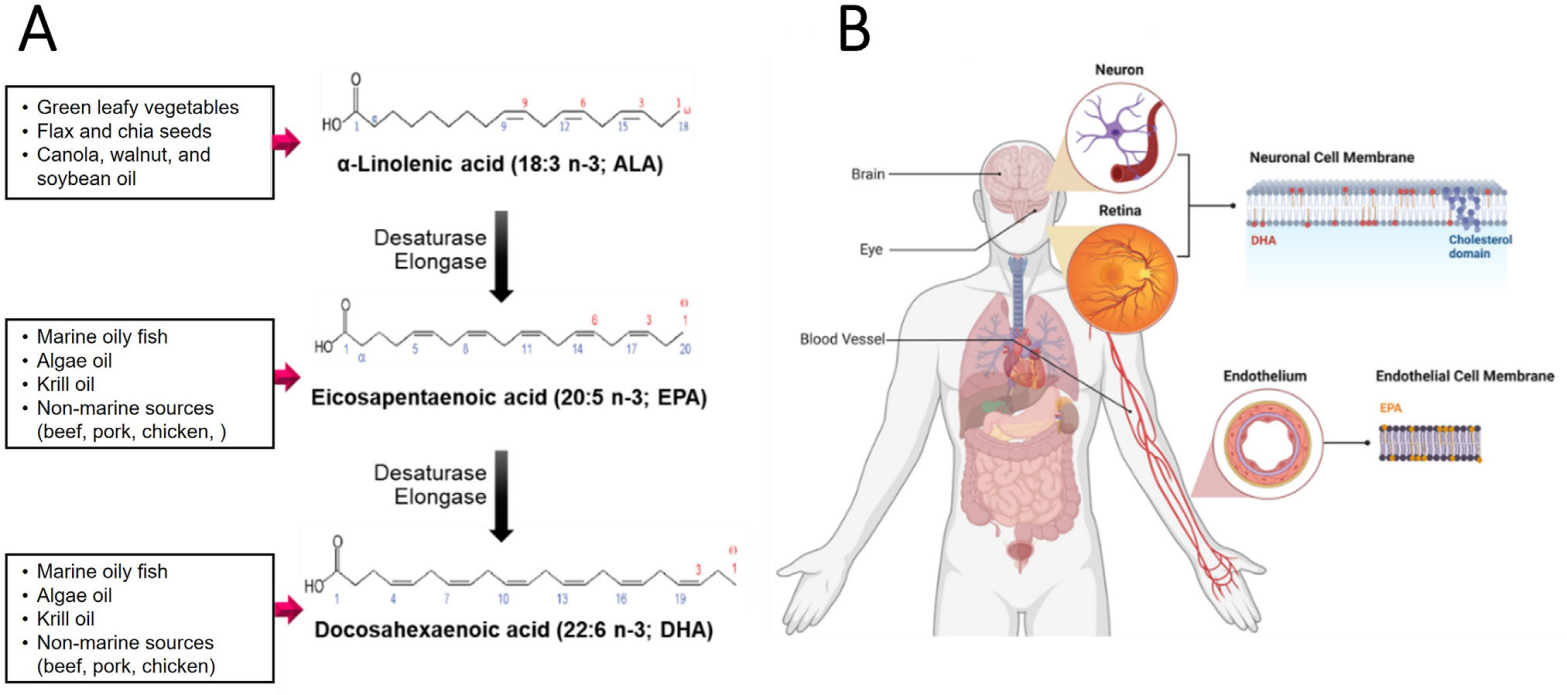
Characteristics of ω-3 PUFAs. (A) Chemical structures and dietary sources of ω-3 PUFAs and pathway for the biosynthetic conversion of ALA to EPA and DHA. Because of the low efficiency of converting ALA to EPA and DHA, it is recommended to consume EPA and DHA from additional dietary sources. Dietary sources of various ω-3 PUFAs are presented in the box on the left. (B) EPA and DHA in anatomical compartments of the human body. ω-3 PUFAs are an important component of the membranes that surround each cell in our body. EPA and DHA are found in cell membranes esterified into phospholipids and other complex lipids. These long-chain ω-3 PUFAs preserve membrane fluidity and inhibit lipid oxidation and membrane cholesterol domain formation. Especially, DHA is highly concentrated in human brain and eyes.

ω-3 PUFAs are important structural components of the cell membrane phospholipid bilayers (Figure 1) [12,13]. Among PUFAs, essential FAs are indispensable for normal growth and development, cellular homeostasis, and various physiological functions. As essential FAs cannot be synthesized in the body, a decrease in the absolute amount results in a deficiency.

ALA, the most common ω-3 PUFA, can be converted into EPA (20:5n–3) and then into DHA (22:6n–3), but the conversion (primarily in the liver) rates are <15% [14,15]. Therefore, consuming EPA and DHA directly from foods or dietary supplements is the only practical way to increase their concentrations in the body (Figure 1A). ALA can be found in vegetable oils, nuts, flax seeds, flaxseed oil, leafy vegetables, and some types of animal fat, especially in grass-fed animals [16,17]. In this review, we differentiated between the 18-carbon ω-3 PUFA (ALA) and the 20- to 22-carbon LC ω-3 PUFAs (EPA and DHA) to clarify their biological effects.

LC ω-3 PUFAs are primarily synthesized by aquatic microalgae and are present in fish, fish oils, and krill oils. When fish consume phytoplankton that have ingested microalgae, ω-3 PUFAs accumulate in their tissues [17]. LC ω-3 PUFAs can also be consumed through nonmarine sources, such as beef, pork, and chicken, wherein these PUFAs are present as part of the membrane phospholipids [18]. However, the content of these PUFAs in animal meat is ∼100 times less than that in fish [19]. Although the consumption of fish, such as salmon, sardines, and mackerel, has been cautioned due to the concern of possibly increasing risk of heavy metal accumulation, there is compelling evidence that the many benefits of fish consumption outweigh any risk [20]. Meanwhile, as of now, the proportion of processed foods or meat in human diet has increased, and the ingestion of fish—the major source of ω-3 PUFAs—has decreased. Thus, it is recommended to take fish oil capsules (either as over-the-counter dietary supplements or pharmaceutical-grade preparations) made from plant microalgae or extracted with an appropriate method [14,16] to replenish the intake of ω-3 PUFAs while minimizing the potential complication described above.

### Biological effects of LC ω-3 PUFAs

LC ω-3 PUFAs are found in the cell membrane and rapidly esterified and incorporated into lipoproteins and membrane phospholipids [12]. Notably, EPA and DHA have different effects on the cellular and molecular mechanisms of atherosclerosis [13,21]. EPA preserves membrane structure and fluidity and normal cholesterol distribution and inhibits membrane lipid oxidation and cholesterol crystal formation [13]. Moreover, EPA produces specialized proresolving mediators (SPMs) [22] and influences signal transduction pathways linked to inflammation and vasodilation [13]. DHA also plays an essential role in the composition and function of the cell membrane, cell signaling, and the production of SPMs [22]. DHA increases membrane fluidity, promotes lipid domain change, and reduces antioxidative properties [21]. Because DHA is highly concentrated in the neuronal membranes (brain) and retina photoreceptors (eyes), it is involved in their development and function (Figure 1) [23]. Despite the mechanistic evidence, it is uncertain whether these distinct actions of EPA and DHA have a significant impact on risk of atherosclerotic cardiovascular disease (ASCVD).

LC ω-3 PUFA supplementation has been reported to reduce CV mortality in large-scale clinical trials involving people with pre-existing CVD or at high CV risk [24]. Dietary intake of EPA and DHA was associated with a reduced risk of CAD mortality [[2], [3], [4]]. A pooled analysis of 17 prospective cohort studies has shown that circulating EPA and DHA concentrations are associated with a lower risk for CV mortality [4]. In addition, LC ω-3 PUFA supplementation in the form of fish oil was associated with the prevention of CVD [2].

In 2018, the AHA suggested consuming seafood 1 to 2 times a week, equivalent to 250 mg/d of EPA and DHA, to prevent CVD [3]. Evidence from epidemiological studies and RCTs recommended the intake of 500 mg/d and 800 to 1000 mg/d of EPA plus DHA for people without or with CVD, respectively [25]. The European Food Safety Authority indicated that daily ingestion of 250 to 500 mg EPA plus DHA reduced risk of CAD and sudden cardiac death [26].

## Evidence of the Role of LC ω-3 PUFAs from a Cardiometabolic Perspective

It has been reported that LC ω-3 PUFAs decrease circulating TG concentrations and have a positive impact on inflammation and vascular endothelial cell function [25]. Intake of LC ω-3 PUFAs lowers plasma TG concentrations by reducing the synthesis of VLDL-1 in human liver [27]. Consuming LC ω-3 PUFAs might also be associated with increased insulin sensitivity and decreased risk of type 2 diabetes [28,29]. Administration of LC ω-3 PUFAs increases their proportions in the cell membrane, helping the synthesis of bioactive mediators that preserve mitochondrial function, alleviate oxidative stress, and protect against CVD-related damage by maintaining ionic homeostasis [30]. LC ω-3 PUFAs also reduce abnormal platelet activation and have a vascular protective effect by acting as a component of the blood vessel wall [31]. Atherosclerosis is a chronic inflammatory condition involving various cell types, such as immune cells, vascular smooth muscle cells, and vascular endothelial cells [32]. LC ω-3 PUFAs may inhibit the development and progression of atherosclerotic lesions by affecting the expression of endothelial cell adhesion molecules, migration and infiltration of monocytes/macrophages, interactions of monocytes/macrophages with T cells, production of proinflammatory cytokines, and proliferation of vascular smooth muscle cells [33]. In addition, LC ω-3 PUFAs are involved in the pathways related to atherosclerotic plaque stability and platelet thrombus formation [33]. Therefore, LC ω-3 PUFAs have pleiotropic effects on vital organs and tissues, which might lead to CV and metabolic benefits (Supplemental Figure 1).

## SPMs Produced from EPA and DHA

Over the past decade, SPMs, including protectins, resolvins, and maresins, have been in the limelight as potent autacoids endogenously produced from enzymatic oxygenation of LC ω-3 PUFAs [22,34]. A methodological study in humans demonstrated increased plasma concentrations of SPMs after intravenous supplementation with EPA and DHA [35]. SPMs have proven to possess anti-inflammatory effects in basic research [30]. They have also emerged as potential regulators of physiologic pathways in the resolution of inflammation and unresolved inflammation [36].

Resolvin D2 and maresin 1 treatment reduced TNF-α–stimulated p65 translocation, superoxide production, and monocyte chemoattractant protein-1 gene expression and inhibited aortic smooth muscle cell migration in cell-based experiments [37]. Treatment with these SPMs reduced neointimal hyperplasia at 14 d in carotid artery-injured mice by 62% and 67%, respectively [37]. Another study using a balloon artery-injured rat model showed that resolvin D1 and protectin D1 decreased neointimal hyperplasia by 37.3% and 31.8%, respectively [38]. Additionally, they attenuated the infiltration of inflammatory cells and mitigated NF-κB activity [38]. The free acid and liposome forms of resolvin D1 alleviated acute inflammation initiated by MI, thereby delaying the onset of heart failure [39]. Brown adipose tissue-derived maresin 2 reduced obesity-induced inflammation partly by promoting macrophages in the liver [40]. Thus, SPMs are likely to have beneficial roles beyond LC ω-3 PUFA derivatives in intermediary metabolism and cell membrane dynamics in the CV system [30], possibly exerting antiatherosclerotic effects by attenuating proinflammatory stimulus [41]. The main mechanisms of SPM actions are presented in Supplemental Figure 2.

## Multifaceted Effects of LC ω-3 PUFAs

**FIGURE 2:**
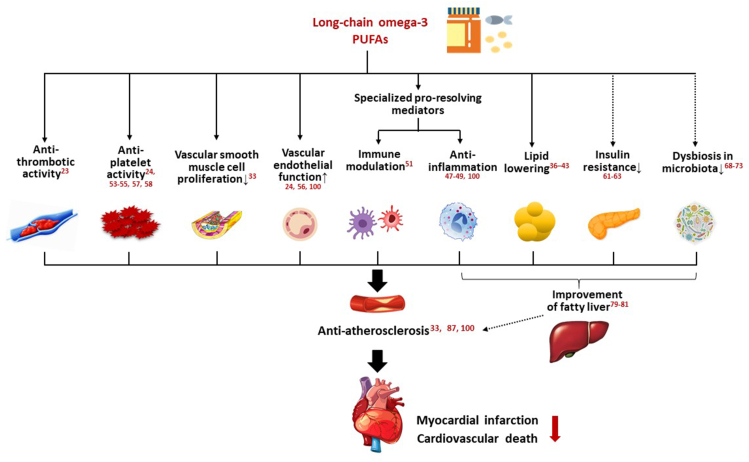
Potential mechanisms of how long-chain ω-3 PUFAs mitigate atherosclerosis and reduce cardiovascular risk.

Potential mechanisms of how LC ω-3 PUFAs mitigate atherosclerosis and reduce CV risk are shown in Figure 2.

### Effects on lipid metabolism

In RCTs involving people with severe hypertriglyceridemia (≥500 mg/dL), a gross weight of 4 g/d ω-3 PUFAs significantly reduced TG concentrations (median changes −52% to −31%) compared with placebo [[42], [43], [44], [45]]. The TG-lowering efficacy might depend on baseline circulating TG concentrations [[42], [43], [44], [45]]. In these trials, a gross weight of 4 g ω-3 PUFAs also decreased non-HDL (median change −18% to −10%), VLDL (median change −41% to −21%), and total cholesterol (median change −16% to −8%) compared with placebo [[42], [43], [44], [45]]. The effects of ω-3 PUFAs on HDL cholesterol (median change −4% to +9%), LDL cholesterol (median change −2% to +49%) [[42], [43], [44], [45]], and apoB (median change −9% to +2%) [44,45] were inconsistent between the studies. Based on these findings, ω-3 ethyl esters, ω-3 carboxylic acids, ω-3 ethyl esters A, and IPE are approved as dietary adjuncts to reduce circulating TG concentrations in adults with severe hypertriglyceridemia (Table 1). In people with moderate hypertriglyceridemia (200 to <500 mg/dL), a gross weight of 4 g/d ω-3 PUFAs also significantly reduced circulating TG concentrations (median change −23% to −22%), whereas it increased LDL cholesterol compared with placebo [[46], [47], [48]].

**TABLE 1:** Information and related studies of prescription ω-3 FAs

Products	EPA and DHA combination			EPA alone	
	Lovaza	Epanova	Omtryg	Vascepa	Epadel
Company	GlaxoSmithKline	AstraZeneca Pharmaceuticals LP	Trygg Pharma, Inc.	Amarin Pharma, Inc.	Mochida Pharmaceuticals Co., Ltd
Chemical structures	ω-3 ethyl esters	ω-3-carboxylic acids	ω-3 ethyl esters A	Ethyl EPA	Ethyl EPA
Contents	EPA 0.465 g and DHA 0.375 g	EPA 0.550 g and DHA 0.200 g	EPA 0.465 g and DHA 0.375 g	Ethyl EPA 0.5 g or 1 g	Ethyl EPA 0.3 g
Daily dose	4 g (EPA 1.86 g and DHA 1.5 g)	2 g (EPA 1.1 g and 0.4 g) or 4 g (EPA 2.2 g and DHA 0.8 g)	4 g (EPA 1.86 g and DHA 1.5 g)	4 g (ethyl EPA 4 g)	1.8 g (ethyl EPA 1.8 g)^1^
Dosage and administration	4 g (4 capsules) once daily or 2 g (2 capsules) twice daily with or without meals	2 g (2 capsules) once daily or 4 g (4 capsules) once daily without regard to meals	4 g (4 capsules) once daily or 2 g (2 capsules) twice daily with meals	2 g (0.5-g 4 capsules or 1-g 2 capsules) twice daily with meals	0.9 g (0.3-g 3 capsules) twice daily or 0.6 g (0.3-g 2 capsules) thrice daily after meals
Inactive gradients	α-tocopherol, gelatin, glycerol, and purified water	α-tocopherol, porcine Type A gelatin, glycerol, sorbitol, and purified water	α-tocopherol, gelatin, glycerol, and purified water	tocopherol, gelatin, glycerin, maltitol, sorbitol, and purified water	α-tocopherol, gelatin, D-sorbitol, glycerin, and parahydroxypenzoate
Approval	US FDA, 2004	US FDA, 2014	US, FDA, 2014	US FDA, 2012	Japan, 1988
Indication for hyperlipidemia	Adjunct to diet to reduce TG concentrations in adults with severe hypertriglyceridemia (≥500 mg/dL)	Hyperlipidemia	—	—	—
Indication for CVD	NA	NA	NA	Adjunct to maximally tolerated statin therapy to reduce risk of MI, coronary revascularization, stroke, and unstable angina requiring hospitalization in adults with TG ≥150 mg/dL and established CVD or DM and ≥2 additional risk factors for CVD	Improvement of ulcer, pain and cold feeling associated with arteriosclerosis obliterans
Clinical trials in severe hypertriglyceridemia (≥500 mg/dL)	Harris et al. [42], Pownall et al. [43]	EVOLVE [44]	Published only in prescribing information (NCT01229566)	MARINE [45]	NA
Clinical trials investigating CV outcomes	SHOT [91], GISSI-Prevenzione [86], GISSI-HF [107], OMEGA [108], ORIGIN [109], FORWARD [110], Risk and prevention [93], ASCEND [29], VITAL [88]	STRENGTH [6]	NA	REDUCE-IT [5]	JELIS [87]

ASCEND, A Study of Cardiovascular Events in Diabetes; CV, cardiovascular; DM, diabetes mellitus; EVOLVE, EpanoVa for lowering very high triglycerides; FORWARD, fish oil research with ω-3 for atrial fibrillation recurrence delaying; GISSI-HF, Gruppo Italiano per lo Studio della Sopravvivenza nell’Infarto miocardico; GISSI-Prevenzione, Gruppo Italiano per lo Studio della Sopravvivenza nell’Infarto miocardico-Prevenzione; JELIS, Japan EPA Lipid Intervention Study; MARINE, multicenter, placebo-controlled, randomized, double-blind, 12-wk study with an open-label extension; NA, not applicable; OMEGA; ORIGIN, Outcome Reduction with an Initial Glargine Intervention; REDUCE-IT, Reduction of Cardiovascular Events with Icosapent Ethyl-Intervention Trial; SHOT, shunt occlusion trial; STRENGTH, Long-Term Outcome Study to Assess Statin Residual Risk Reduction with EpaNova in High Cardiovascular Risk Patients with Hypertriglyceridemia; VITAL, Vitamin D and ω-3 Trial.

1 Can be increased to 2.7 g/d if TG concentrations remain abnormal.

LC ω-3 PUFA supplementation can lead to an increase in LDL cholesterol concentrations. However, TG lowering by LC ω-3 PUFAs may reduce the cholesteryl ester transfer protein-mediated transfer of TG from VLDL to LDL, thereby increasing the LDL particle size. It leads to an increase in less atherogenic large buoyant LDL particles rather than atherogenic small dense LDL particles [49]. In addition, LC ω-3 PUFAs can reduce TRLs and their remnants. Emerging evidence suggests that an increase in TRLs and their remnants contributes to risk of ASCVD and partly explains residual CV risk after statin therapy [50]. Therefore, the beneficial effects of LC ω-3 PUFAs on lipid metabolism can contribute to a reduction in atherosclerotic risk.

### Anti-inflammation

Several mechanisms whereby LC ω-3 PUFAs play a beneficial role in the inflammatory process have been postulated. One study reported that purified EPA produced EPA-rich HDL [51]. This reconstituted form of HDL contained EPA-phosphatidylcholine, which had antiatherogenic properties and decreased vascular cell adhesion molecule-1 expression [51]. Moreover, the reconstituted HDL enhanced cholesterol efflux and produced resolvin E3 and 18-hydroxy-EPA, EPA-derived anti-inflammatory metabolites [51]. The increased flux of free FAs associated with metabolic impairments, such as obesity, insulin resistance, and type 2 diabetes, causes endothelial dysfunction by enhancing NF-κB-related inflammatory pathways [52]. DHA reduced cytokine-induced expression of endothelial adhesion molecules and decreased secretion of IL-6 and IL-8 in endothelial cells [52]. In a randomized, crossover study, DHA treatment significantly reduced the concentrations of inflammatory markers, such as IL-18, compared with EPA treatment [53]. LC ω-3 PUFAs also exhibited anti-inflammatory effects by binding to membrane phospholipids and reducing AA, known as a representative product of ω-6 PUFAs [54].

### Immunomodulation

LC ω-3 PUFAs modulate the immune response by converting them into SPMs [30]. SPMs are produced by the oxidation of essential FAs, including EPA, DHA, and DPA. They are classified as resolvin, protectin, and maresin. LC ω-3 PUFA administration can increase SPMs, which have protective effects on the CV and CNSs [30]. In an animal model of MI, bioactive mediators converted from LC ω-3 PUFAs, such as resolvin E1 and resolvin D1, showed cardioprotective effects by reducing macrophage infiltration and inflammatory mediators [55]. Neuroprotectin D1 and resolvin D2 also enhanced cell survival signaling pathways, maintained blood-brain barrier integrity, and promoted angiogenesis during acute CV ischemia [56,57].

### Antiplatelet activity

LC ω-3 PUFAs affect thrombotic mechanisms, including platelet activation and aggregation. On top of aspirin and clopidogrel treatment, EPA decreased platelet activation and adhesion, thromboxane A2 synthesis, and the concentrations of plasminogen activator inhibitor-1 [58].

A previous human study reported that EPA supplementation decreased thromboxane A2 concentrations by 58%, leading to increased formation of the inactive thromboxane A3 [59]. LC ω-3 PUFA supplementation also increased prostaglandin I3 and inactive thromboxane A3 concentrations, thereby improving the prostaglandin I/thromboxane A balance, which had cardioprotective and antithrombotic effects [60]. Thus, LC ω-3 PUFAs might lead to CV benefits by reducing platelet function and hemostasis in conjunction with lowering TG concentrations, improving endothelial function, and alleviating inflammation [61,62]. It should be noted that a highly purified form of DHA might be more antithrombotic than EPA [63]. In addition, men may benefit more from EPA whereas women are more responsive to DHA for platelet aggregation [64]. This finding suggests that interactions between sex hormones and LC ω-3 PUFAs may reduce platelet aggregation differentially in healthy individuals [64].

### Effects on glucose homeostasis

LC ω-3 PUFAs could benefit people with insulin resistance and glucose intolerance via multiple mechanisms because the TG-lowering efficacy is associated with improvements in glucose metabolism [65]. In a meta-regression of 45 RCTs, ω-3 PUFA supplementation improved glucose regulation and reduced TNF-α and IL-6 concentrations as well as induced favorable changes in lipid profiles [66]. An RCT involving healthy older adults showed that intake of ω-3 PUFAs increased muscle mass and improved muscle function [67], which is known to be positively associated with glucose homeostasis. In a *db/db* mice study, 1-wk treatment with EPA without DHA alleviated insulin resistance, decreased fasting insulin and glucose concentrations, and improved glucose intolerance [68]. Treatment with EPA alone also improved pancreatic β-cell function, reduced liver TGs with enhanced gene expression in hepatic FA oxidation, and altered microbiota composition [68].

Although many studies have demonstrated various benefits of LC ω-3 PUFA supplementation, its long-term effects on the prevention or treatment of type 2 diabetes remain controversial. Observational studies have suggested both positive and negative effects of LC ω-3 PUFAs on glucose metabolism and risk of type 2 diabetes [69,70]. In a meta-analysis of 83 RCTs involving 121,070 adults at any risk of type 2 diabetes, intake of ω-3, ω-6, and total PUFAs (mainly LC ω-3 PUFAs; mean dose: 2.0 g/d, mean duration: 33 mo) had little or no effect on incident type 2 diabetes and glucose metabolism (HbA1c, fasting plasma glucose, fasting insulin, and the HOMA-IR) [71]. Given the results and cardiometabolic benefits of LC ω-3 PUFAs, large-scale RCTs are needed to elucidate the effects on glucose metabolism.

### Impact on gut microbiota

In a study with *db/db* mice, EPA and DHA supplementation reduced the abundance of the LPS-containing *Enterobacteriaceae.* At the same time, it instead increased that of beneficial *Bifidobacterium*, *Lactobacillus*, and SCFA-producing species [72]. The gut microbiome alterations were accompanied by shifts in the metabolome, including glutamate, bile acids, propionic and butyric acids, and LPS, which subsequently relieved pancreatic β-cell apoptosis, suppressed hepatic gluconeogenesis, and facilitated the secretion of glucagon-like peptide-1 [72].

A study using data from 876 twins with 16S microbiome and ω-3 PUFAs reported that serum concentrations of total ω-3 PUFAs and DHA were significantly correlated with microbiome α-diversity (Shannon index) after adjusting for confounders [73]. These associations remained significant after adjusting for dietary fiber intake. An RCT with a sardine diet (100 g sardines for 5 d/wk for 6 mo providing ∼3 g daily of an EPA and DHA combination) in people with type 2 diabetes reported a significant decrease in the *Firmicutes*-to-*Bacteroidetes* ratio compared with control [74]. Eight weeks of ω-3 PUFA supplementation in healthy volunteers caused a consistent and reversible increase in the SCFA-producing intestinal microbiome [75]. However, a lack of significant changes in microbial diversity was found [75], consistent with a mice study in which there was either no or only a tiny change in α-diversity [76]. Of note, short-term dietary interventions could not alter the dominant interindividual variation in the gut microbiome [77]. Taken together, LC ω-3 PUFAs are likely to attenuate hyperglycemia and insulin resistance by affecting the gut microbiome and metabolites linking the gut to adipose tissue, liver, and pancreas. Therefore, LC ω-3 PUFA supplementation might be helpful restore glucose homeostasis through favorable changes in the gut–organs axis.

### Effects on fatty liver

The novel term metabolic dysfunction-associated fatty liver disease (MAFLD) has been proposed to replace nonalcoholic fatty liver disease (NAFLD) [78]. The concept of NAFLD has several pitfalls. NAFLD is a diagnosis of exclusion (thus not based on positive diagnostic criteria that are more definitive), and the histologic confirmation of nonalcoholic steatohepatitis (NASH) can be difficult in some cases because of significant intra- and interobserver variability [78]. A recent review has advocated redefining this condition as MAFLD by raising concerns about the current definition of NAFLD [79].

Currently, the principal therapy for NAFLD involves dietary and lifestyle modifications to lose body weight while improving liver steatosis and inflammation [80,81]. Pioglitazone, vitamin E, or their combination therapy can be used in people with NASH; however, there are no drugs with sufficient evidence. Some studies have proposed that dietary ω-3 PUFAs improve insulin resistance by regulating mitochondrial function and mediating anti-inflammatory effects [82,83]. In a systemic review and meta-analysis of 17 human studies that evaluated the effects of marine ω-3 PUFAs on NAFLD [84], 12 studies reported a decrease in liver fat or other markers of NAFLD after ω-3 PUFA supplementation. Five studies did not show any benefit in liver enzyme activities, which seemed to be owing to the relatively short duration of treatment, inadequate adherence, and methodological problems [84]. Thus, LC ω-3 PUFAs can be used as adjuncts to lifestyle modifications for treating patients with MAFLD. However, further well-designed RCTs are warranted.

## Effect of LC ω-3 PUFAs on CV Events in Large-Scale RCTs

Although several prospective observational studies have reported that consuming LC ω-3 PUFAs prevented incident CAD [85], RCTs have shown discrepant results for CVD. In the Gruppo Italiano per lo Studio della Sopravvivenza nell’Infarto miocardico (GISSI)-Prevenzione trial, supplementation with 1 g/d of ω-3 PUFAs therapy was associated with early protection from sudden cardiac death in patients who had a myocardial infarction [86]. In Japan EPA Lipid Intervention Study (JELIS) involving Japanese people with hypercholesterolemia, additional administration of 1.8 g EPA for 5 y reduced the incidence of major coronary events compared with a statin alone (2.8% compared with 3.5%) [87]. JELIS conducted an open-label, blinded endpoint evaluation. The mean LDL cholesterol concentrations were 182 mg/dL at baseline but were not related to the reduction in major coronary events. In A Study of Cardiovascular Events in Diabetes (ASCEND) involving people with type 2 diabetes, administrating 1 g of ω-3 PUFAs for 7 y did not result in any difference in the incidence of any severe CVD compared with placebo [29]. In Vitamin D and ω-3 Trial (VITAL), an RCT involving 25,871 adults with a 2 × 2 factorial design of vitamin D3 and 1 g/d of marine ω-3 PUFAs, MACE did not differ between the ω-3 PUFA and placebo groups during a median 5.3 y (HR: 0.92; 95% CI: 0.80, 1.06; *P* = 0.24) [88]. In the analysis of key secondary endpoints, HRs for total myocardial infarction, stroke, and CV death were 0.72 (95% CI: 0.59, 0.90), 1.04 (95% CI: 0.83, 1.31), and 0.96 (95% CI: 0.76, 1.21), respectively [88].

In REDUCE-IT, which involved people with established CVD or type 2 diabetes plus ≥1 additional CV risk factor, taking 4 g/d IPE significantly lowered risk of major ischemic events by 25% compared with a mineral oil placebo [5]. STRENGTH administered an ω-3 carboxylic acid formulation (EPA and DHA) at a dose of 4 g/d in people with atherogenic dyslipidemia and established ASCVD or high CV risk. However, STRENGTH was terminated early because it did not show any benefits compared with a corn oil placebo in the interim evaluation [6]. Besides the types or doses of LC ω-3 PUFAs, there were differences in study populations and comparators between the 2 studies. The proportions of people with secondary prevention were 70.7% and 55.6% in REDUCE-IT and STRENGTH, respectively, at baseline. REDUCE-IT used mineral oil as a comparator, which increased LDL cholesterol (+10%), non-HDL cholesterol (+9%), apoB (+8%), and CRP (+32%) from baseline [89]. In contrast, corn oil used as a comparator in STRENGTH showed neutral effects on lipid profiles and a slight decrease in CRP (−6%) [89]. In the Effect of Vascepa on Improving Coronary Atherosclerosis in People With High Triglycerides Taking Statin Therapy (EVAPORATE), which used similar eligibility criteria to REDUCE-IT, administration of 4 g/d IPE significantly reduced plaque volume assessed by coronary CT angiography compared with placebo over 18 mo (−17% compared with +109%) [90]. The median TG concentrations were 259.1 mg/dL at baseline. EVAPORATE also showed no difference in plaque progression between mineral oil and cellulose-based placebos, partly explaining the CV benefit observed in REDUCE-IT [90]. However, there are concerns about normalizing baseline plaque volume and blind assessment of plaque quantification. Therefore, additional evidence is needed to support the association of LC ω-3 PUFAs with preventing CVD.

To provide a precise estimate of treatment effects, we conducted a meta-analysis of RCTs investigating the effects of LC ω-3 PUFA supplementation on CV outcomes. We searched MEDLINE (via PubMed) up to March 2022 to identify eligible studies that reported outcomes of interest with ≥500 participants and ≥1 y of follow-up. We included 17 studies involving 143,410 participants ranging from 546 to 25,871 in each study. Two studies used EPA alone [5,87], and the remaining studies used EPA and DHA combinations in the intervention group. All except 3 studies [86,87,91] used placebos in the control group. The VITAL Rhythm Study [92] was an ancillary study of VITAL [88]. Table 2 summarizes the baseline characteristics of included studies.

**TABLE 2:** Baseline characteristics of major randomized controlled trials investigating the effects of ω-3 FAs on cardiovascular outcomes

Reference	Year	Study population	*n*^1^ (M/F)^2^	Age, y^3^	BMI, kg/m^2^^3^	Intervention; EPA/DHA dose, mg/d	Intervention; other agents	Control	Mean follow-up, y	DM, *n* (%)	HTN, *n* (%)^4^	Statin use, *n* (%)	Prior CAD, *n* (%)	Prior stroke, *n* (%)
SHOT [91] 5	1996	Undergoing CABG	610 (531/79)	60 (9)	25.3 (2.8)	4000; 2040/1280	Aspirin, warfarin	No placebo	1.0	43 (7)	137 (22)	NA	610 (100)	NA
GISSI-Prevenzione [86] 5	1999	Recent MI	11,324 (9659/1665)	59 (11)	26.5 (3.7)	1000; ≈394/472 (850–882 in total)	Vitamin E	No placebo	3.5	1683 (15)	4026 (36)	NA	11,324 (100)	NA
SOFA [111]	2006	ICDs for VT or VF	546 (459/85)	62 (14)	26.9 (4.9)	2000; 464/335	None	High-oleic acid sunflower oil	1.0^6^	87 (16)	177 (32)	NA	384 (70)	43 (8)
JELIS [87]	2007	Hypercholesterolemia with statin treatment	18,645 (5859/12,786)	61 (9)	24.0 (3.0)	1800; 1800/0	None	No placebo	4.6	3040 (16)	6611 (35)	18,003 (97)	3664 (20)	NA
GISSI-HF [107]	2008, 2013	HF	6975 (5459/1516)	67 (11)	27.0 (5.0)	1,000; ≈394/472 (850–882 in total)	None	Olive oil	3.9^6^	1974 (28)	3809 (55)	1579 (23)	NA	346 (5)
Alpha omega [112] 5	2010	MI	4837 (3783/1054)	69 (6)	27.8 (3.9)	400; 226/150	None	Margarine or ALA only	3.4^6^	1014 (21)	4340 (90)	NA	4837 (100)	345 (7)
DOIT [113] 5	2010	Hypercholesterolemia	563 (563/0)	70 (3)	NA	2400; 1176/840	Diet counseling	Corn oil	3.0	82 (15)	158 (28)	NA	NA	NA
OMEGA [108]	2010	Recent MI	3851 (2841/977)	64 (NA)	27.4^6^ (NA)	1000; 460/380	None	Olive oil	1.0	1032 (27)	2538 (66)	3113 (81)	3851 (100)	209 (5)
SU.FOL.OM3 [103] 5	2010	MI, unstable angina, ischemic stroke	2501 (1987/514)	61 (9)	27.2^6^ (NA)	600; 400/200	B vitamins	Placebo (not reported)	4.2	NA	NA	NA	1863 (74)	638 (26)
ORIGIN [109] 5	2012	High CV risk with IFG, IGT, or early DM	12,611 (8150/4386)	64 (8)	29.8 (5.3)	1000; 465/375	Insulin glargine	Olive oil	6.2^6^	11,081 (88)	9962 (79)	6739 (53)	NA	NA
FORWARD [110]	2013	AF	586 (321/265)	66 (11)	NA	1000; NA/NA (850–882 in total)	None	Olive oil	1.0	74 (13)	524 (89)	NA	67 (11)	27 (5)
Risk and Prevention [93]	2013	Multiple CV risk factors, ASCVD	12,513 (7687/4818)	64 (9)	29.4 (5.0)	1000; 410/456; (850–882 in total)	None	Olive oil	5.0^6^	7494 (60)	10,577 (85)	5138 (41)	NA	594 (5)
COS [114] 5	2014	Age-related macular degeneration	4203 (1815/2388)	74^6^ (NA)	NA	1000; 650/350	Lutein plus zeaxanthin	AREDS supplement^7^	4.8^6^	546 (13)	NA	1850 (44)	405 (10)	211 (5)
ASCEND [29] 5	2018	DM but without ASCVD	15,480 (9684/5796)	63 (9)	30.8 (6.3)	1000; 460/380	Aspirin	Olive oil	7.4	14,569 (94)	9533 (62)	11,653 (75)	0	0
REDUCE-IT [5]	2019	Established CVD or DM and risk factors	8179 (5822/2357)	64^6^ (NA)	30.8^6^ (NA)	4000; 4000/0	None	Mineral oil	4.9^6^	4787 (59)	NA	8145 (100)^8^	NA	NA
VITAL [88] 5, VITAL Rhythm Study [92] 5^,^^9^	2019	Men ≥50 y, women ≥55 y	25,871 (12,786/13,085)	67 (7)	28.1 (5.7)	1000; 460/380	Vitamin D_3_	Placebo (not reported)	5.3^6^	3549 (14)	12,791 (49)	8890 (34)	0	0
OMEMI [95]	2020	Elderly with recent MI	1027 (720/294)	75 (4)	27.0 (10.0)	1800; 930/660	None	Corn oil	2.0	210 (20)	611 (59)	978 (95)	1027 (100)	NA

STRENGTH [6]	2020	Treated with statin and high CV risk	13,078 (8510/4568)	63 (9)	32.2 (5.7)	4000; 2200/800	None	Corn oil	3.5^6^	9170 (70)	11,420 (87)	13,078 (100)	6035 (46)	1048 (8)

AF, atrial fibrillation; AREDS, age-related eye disease study; ASCEND, A Study of Cardiovascular Events in Diabetes; ASCVD, atherosclerotic cardiovascular disease; CABG, coronary artery bypass graft; COS, Cardiovascular Outcome Study; CV, cardiovascular; DM, diabetes mellitus; DOIT, diet and ω-3 intervention trial; FORWARD, fish oil research with ω-3 for atrial fibrillation recurrence delaying; GISSI-HF, Gruppo Italiano per lo Studio della Sopravvivenza nell’Infarto miocardico-heart failure; GISSI-Prevenzione, Gruppo Italiano per lo Studio della Sopravvivenza nell’Infarto miocardico-Prevenzione; HF, heart failure; HTN, Hypertension; ICD, implantable cardioverter defibrillators; IFG, impaired fasting glucose; IGT; impaired glucose tolerance; JELIS, Japan EPA Lipid Intervention Study; NA, not applicable; OMEGA; OMEMI, ω-3 FAs in elderly patients with MI; ORIGIN, Outcome Reduction with an Initial Glargine Intervention; REDUCE-IT, Reduction of Cardiovascular Events with Icosapent Ethyl-Intervention Trial; SHOT, shunt occlusion trial; SOFA, study on ω-3 FAs and ventricular arrhythmia; STRENGTH, Long-Term Outcome Study to Assess Statin Residual Risk Reduction with EpaNova in High Cardiovascular Risk Patients with Hypertriglyceridemia; SU.FOL.OM3, supplémentation en folates et ω-3; VF, ventricular fibrillation; VITAL, Vitamin D and ω-3 Trial; VT, ventricular tachycardia.

1 Number of randomly assigned participants.

2 Number of analyzed participants.

3 Values are means (standard deviations).

4 Hypertension or treated with antihypertensive agents.

5 Two-by-two factorial design.

6 Values are median.

7 AREDS supplement included ascorbic acid, vitamin D, β-carotene, zinc, and copper.

8 Data from 34 participants were missing.

9 The VITAL Rhythm Study was an ancillary study of the VITAL.

We estimated RRs for 3-point MACE (defined as fatal or nonfatal MI, fatal or nonfatal stroke, and CV death), all-cause mortality, hospitalization for heart failure (HHF), and new-onset atrial fibrillation using a random-effects model. For CV death, JELIS included coronary death [87]. We also conducted subgroup analyses and meta-regressions to assess whether the effects of LC ω-3 PUFAs varied according to the important factors related to its efficacy as follows.

1) Types of LC ω-3 PUFAs: EPA and DHA combination or EPA alone
2) Doses of LC ω-3 PUFAs: EPA plus DHA and EPA
3) Comparators: corn oil, mineral oil, olive oil, others, or no placebo
4) Types of prevention: primary prevention, secondary prevention, or both

Statistical analyses were performed using R version 4.0.4. *P* values < 0.05 were considered statistically significant for the outcomes and tests for heterogeneity.

### Three-point MACE and its components

**FIGURE 3:**
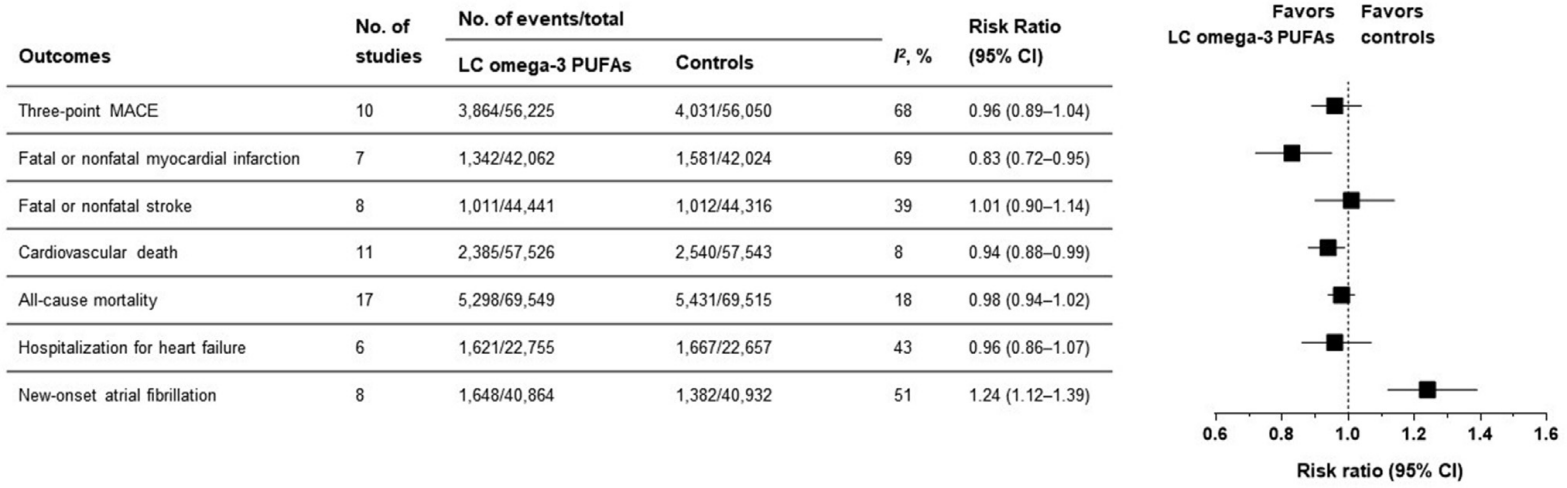
Risks of cardiovascular outcomes and all-cause mortality in people randomly assigned to LC ω-3 PUFA supplementation compared with controls. LC, long-chain; MACE, major adverse cardiovascular events.

LC ω-3 PUFA supplementation did not affect risk of MACE compared with controls (RR: 0.96; 95% CI: 0.89, 1.04; *P* = 0.366) (Figure 3). There was substantial heterogeneity across the studies (*I*^2^ = 68%, *τ*^2^ = 0.010, *P* = 0.001). When assessing its components, LC ω-3 PUFAs led to a 17% and 16% RR reduction in fatal or nonfatal MI (RR: 0.83; 95% CI: 0.72, 0.95; *P* = 0.010) and CV death (RR: 0.94; 95% CI: 0.88, 0.99; *P* = 0.029) (Figure 3). The number needed to treat (NNT) was 175 and 373, respectively. LC ω-3 PUFA supplementation had no significant effect on fatal or nonfatal stroke (RR: 1.01; 95% CI: 0.90, 1.14; *P* = 0.823) (Figure 3).

**FIGURE 4:**
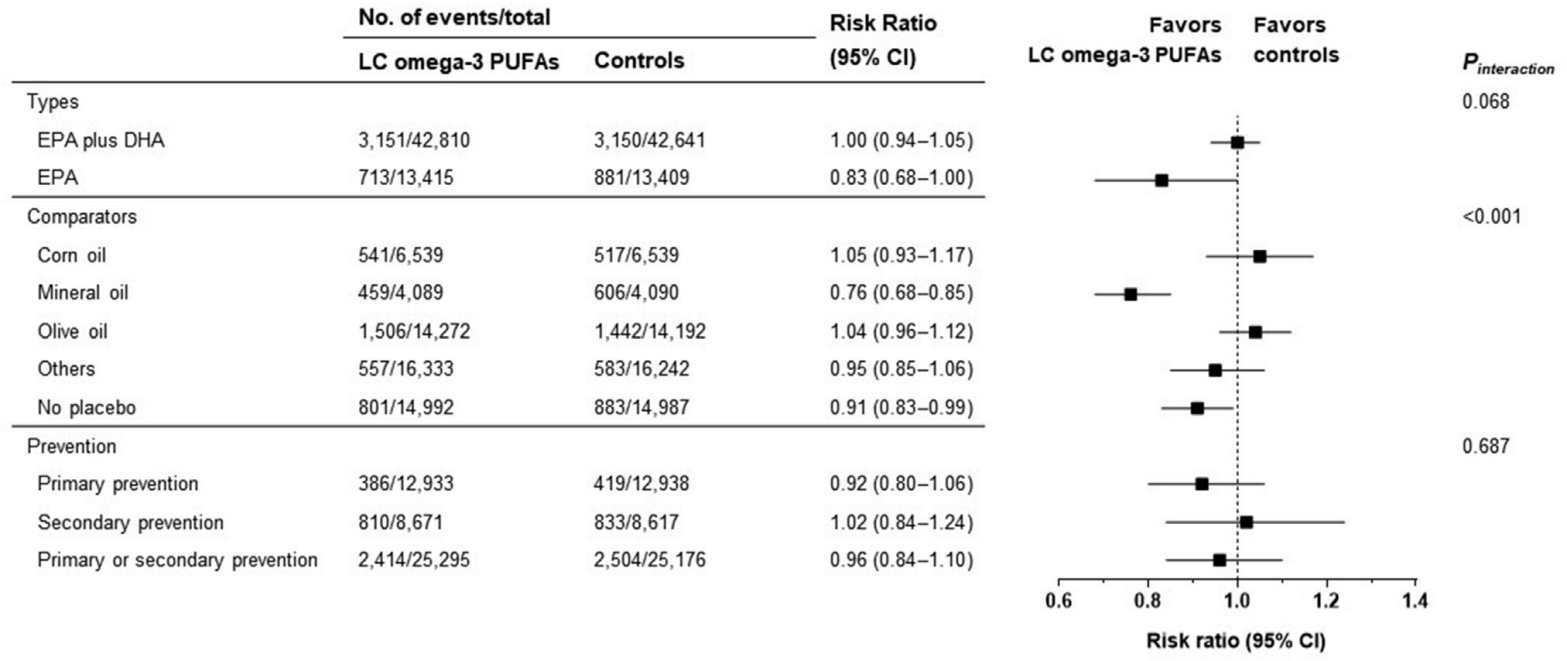
Subgroup analyses for 3-point major adverse cardiovascular events by type and comparator of LC ω-3 PUFAs and study populations. LC, long-chain.

**FIGURE 5:**
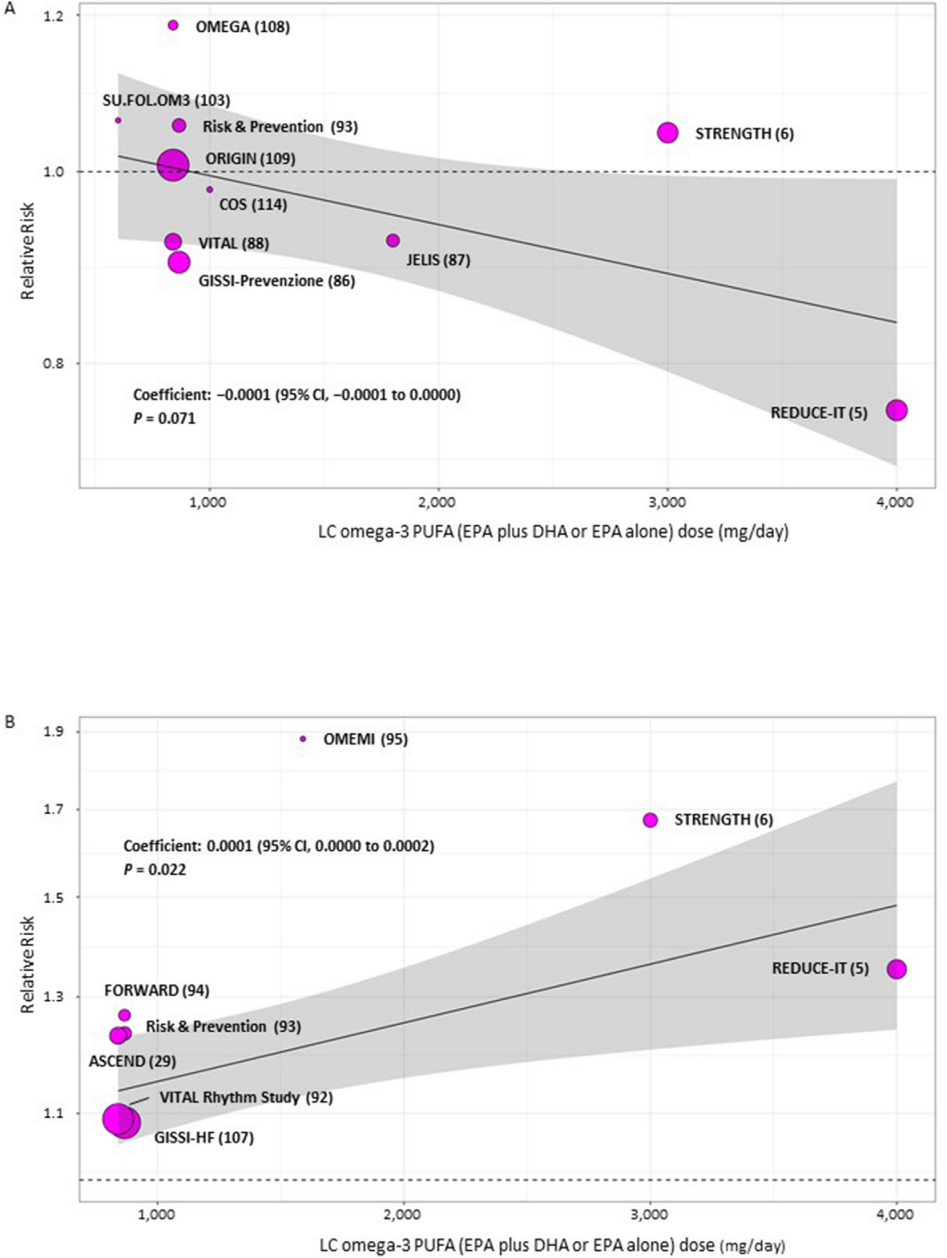
Dose–response association of LC ω-3 PUFAs with (A) 3-point major adverse cardiovascular events and (B) new-onset atrial fibrillation. . ASCEND, A Study of Cardiovascular Events in Diabetes; COS, Cardiovascular Outcome Study; FORWARD, fish oil research with ω-3 for atrial fibrillation recurrence delaying; GISSI-HF, Gruppo Italiano per lo Studio della Sopravvivenza nell’Infarto miocardico-heart failure; GISSI-Prevenzione, Gruppo Italiano per lo Studio della Sopravvivenza nell’Infarto miocardico-Prevenzione; JELIS, Japan EPA Lipid Intervention Study; LC, long-chain; OMEGA; OMEMI, ω-3 FAs in elderly patients with MI; ORIGIN, Outcome Reduction with an Initial Glargine Intervention; REDUCE-IT, Reduction of Cardiovascular Events with Icosapent Ethyl-Intervention Trial; STRENGTH, Long-Term Outcome Study to Assess Statin Residual Risk Reduction with EpaNova in High Cardiovascular Risk Patients with Hypertriglyceridemia; SU.FOL.OM3, supplémentation en folates et ω-3; VITAL, Vitamin D and ω-3 Trial.

In subgroup analyses, there were no differences in MACE according to the types of LC ω-3 PUFAs and prevention (Figure 4, Supplemental Figure 3). However, a significant difference was found in MACE by the comparators (*P* < 0.001), possibly mainly driven by REDUCE-IT [5], which used a mineral oil placebo in the control group (Figure 4, Supplemental Figure 3). Meta-regression analysis showed that EPA (Supplemental Figure 4), but not EPA plus DHA (Figure 5A), showed a dose-dependent decrease in MACE.

### All-cause mortality

LC ω-3 PUFA supplementation had no significant effect on risk of all-cause mortality (RR: 0.98; 95% CI: 0.94, 1.02; *P* = 0.274) (Figure 3, Supplemental Figure 5). There was no significant heterogeneity across the studies. There was no subgroup difference or dose response for all-cause mortality.

### HHF

Risk of HHF was not reduced with LC ω-3 PUFA supplementation (RR: 0.96; 95% CI: 0.86, 1.07; *P* = 0.431) (Figure 3, Supplemental Figure 6). There was no significant heterogeneity across the studies. Although LC ω-3 PUFAs reduced risk of HHF by 32% (RR: 0.68; 95% CI: 0.53, 0.88) in Risk and Prevention trial [93], a sensitivity analysis excluding this study did not alter the direction of the treatment effect (RR: 0.99; 95% CI: 0.93, 1.06). No subgroup difference or dose response was found for HHF.

### New-onset atrial fibrillation

LC ω-3 PUFA supplementation significantly increased risk of new-onset atrial fibrillation (RR: 1.24; 95% CI: 1.12, 1.39; *P* < 0.001), with moderate heterogeneity across the studies (*I*^2^ = 51%, *τ*^2^ = 0.011, *P* = 0.047) (Figure 3, Supplemental Figure 7). The number needed to harm was 153. Meta-regression analysis showed that a significant dose response was observed in new-onset atrial fibrillation with EPA plus DHA supplementation (*P* = 0.022) (Figure 5B). This result was consistent with a previous study finding of a dose-related increase in atrial fibrillation risk over an ω-3 PUFA supplementation range of 1 g/d to 4 g/d [94]. In people with established CVD or multiple CV risk factors, LC ω-3 PUFAs increased risk of atrial fibrillation [5,6,95]. Given the NNT for 3-point MACE was 175 in our study, these findings suggest that the use of LC ω-3 PUFAs should be tailored based on risk of ASCVD and atrial fibrillation. Of note, EPA did not show such a relationship (*P* = 0.087) (Supplemental Figure 8).

Accumulating evidence suggests that ω-3 PUFAs can affect a new class of ion channels called PIEZO1 that functions as a mechanical sensor in cellular membranes [96]. This channel involves multiple biological processes, including mechanical stress-induced signaling in atrial cells such as fibroblasts [97]. PIEZO1 activity in atrial fibroblasts increased in people with atrial fibrillation, suggesting that PIEZO1 might contribute to the structural and electrical remodeling of the atrium [98]. The administration of ω-3 PUFAs may affect PIEZO1 activity, prolonging action potential duration and increasing the propensity for delayed after depolarizations that trigger atrial fibrillation [97].

### Other adverse events

There have been questions about whether ω-3 PUFAs increase risk of bleeding. LC ω-3 PUFAs revealed no excess risks of bleeding or other serious adverse events in the VITAL study [88]. In REDUCE-IT, there was a slightly higher rate of minor bleeding with IPE compared with the placebo [5]. However, no significant increase in fatal bleeding, such as intracranial hemorrhage or gastrointestinal bleeding, was observed in the IPE group [5]. In STRENGTH, treatment-emergent adverse events were more common in the LC ω-3 PUFA group than in the corn oil placebo group (22.2% compared with 12.9%) [6]. Gastrointestinal adverse events also occurred more frequently in the LC ω-3 PUFA group than in the control group (24.7% compared with 14.7%) [6]. However, there were no differences in the rates of bleeding events between the 2 groups [6]. The overall adverse events were similar between the LC ω-3 PUFAs and placebos groups in REDUCE-IT and STRENGTH [5,6].

### Implications

Our meta-analysis demonstrates that LC ω-3 PUFA supplementation reduces risk of CV death and myocardial infarction in a dose-dependent manner. However, it is noteworthy that the CV benefits from LC ω-3 PUFA supplementation differed according to the comparators. LC ω-3 PUFA supplementation had no significant effect on all-cause mortality and HHF. However, LC ω-3 PUFA treatment significantly increased risk of new-onset atrial fibrillation.

Differences in various factors, including the types or doses of LC ω-3 PUFAs, control groups, and study populations, could affect the inconsistent results of LC ω-3 PUFA supplementation. To investigate the role of these factors, we conducted meticulous meta-analyses using strict eligibility criteria of the RCTs involving LC ω-3 PUFAs.

The conflicting CV effects of LC ω-3 PUFAs could arise from different comparators. In REDUCE-IT [5] and its substudies [[99], [100], [101]], IPE robustly reduced ischemic events across a broad range of populations. The main criticism of the REDUCE-IT has been that using a mineral oil placebo might contribute to the beneficial effects of IPE [89]. A cohort study mimicking trial designs showed that the contrasting results of REDUCE-IT and STRENGTH could partly be explained by different effects of comparators on the lipid profile and CRP concentrations [89]. However, the CV benefits of IPE in REDUCE-IT were consistent regardless of the background statin therapy, indicating that lipophilicity of statins or undesirable interaction of statins with mineral oil was not the primary driver of IPE’s clinical efficacy [102].

In addition, EPA and DHA have different chemical properties and might have distinct effects on cell membrane structure, lipid oxidation, inflammatory markers, and endothelial function related to atherosclerosis [21]. Although it is unclear whether DHA may diminish or negate the benefits of EPA, the effect of combined treatment of DHA with EPA needs to be assessed further.

Circulating and tissue concentrations of ω-3 PUFAs at baseline might also be crucial in determining the effects of ω-3 PUFA supplementation on CV events [7,9]. However, clinical studies of ω-3 PUFA therapy often provided insufficient information of PUFA concentrations in the blood or tissue, which may contribute to discordant findings. In STRENGTH [6], the circulating EPA concentration was 89.6 μg/mL at the end of the study, which is much lower than the EPA concentrations in JELIS (169 μg/mL) [87] and REDUCE-IT (144.0 μg/mL) [5]. The major shortcoming of previous studies is that the authors did not measure circulating concentrations of ω-3 PUFAs at baseline and at follow-up. Without these measurements, it is difficult to evaluate the effects of an intervention on changes in ω-3 PUFA concentrations and health outcomes. Future studies should provide information on ω-3 PUFA status by measuring serum [5] or plasma concentrations [6], percentage of total red blood cell FAs [6], percentage of total plasma lipids [103], or the balance of ω-3 highly unsaturated FAs [7].

Thus, there are several limitations and caveats in the interpretation of studies on LC ω-3 PUFAs. First, the effects of circulating ω-3 PUFA concentrations on each outcome were not examined. Second, background diets, including fish and fish oils, may affect the effectiveness of LC ω-3 PUFA supplementation [7,9]. Third, ω-6 PUFA intake might influence the effects of LC ω-3 PUFAs by altering the conversion and storage of ω-3 PUFAs [7,9].

## Use of LC ω-3 PUFAs from a Clinical Perspective

In 2019, the AHA and the European Society of Cardiology released guidelines that LC ω-3 PUFAs (EPA and DHA combination or EPA alone) can be prescribed to decrease circulating TG concentrations and reduce residual CV risk on top of statin treatment [104,105]. JELIS showed that 1.8 g of EPA ethyl ester effectively reduced MACE in Japanese people with hypercholesterolemia [87]. In RECUCE-IT involving high-risk people on statin therapy, the use of 4 g/d IPE for improving risk of severe ASCVD in people with high TG is supported by a 25% reduction in MACE [5]. On the other hand, STRENGTH, which evaluated the effect of 4 g/d ω-3 carboxylic acids (EPA and DHA) in people with high TG concentrations and low HDL cholesterol concentrations on statin treatment, failed to prove the CV benefits [6]. Moreover, an increased risk of atrial fibrillation, particularly noticed in REDUCE-IT [5] and STRENGTH [6], requires a balanced approach to LC ω-3 PUFA therapy CVD prevention and potentially harmful effects. However, LC ω-3 PUFAs generally have good safety and tolerability. To date, <5% of participants have discontinued taking LC ω-3 PUFAs due to adverse events in major RCTs [5,6]. Current evidence suggests that 1.8 g/d of EPA for East Asians and 4 g/d of IPE for Western people reduce the residual CV risk. This recommendation stems from the result that a plasma EPA concentration (170 μg/mL) from 1.8 g/d of EPA in a Japanese population was similar to that obtained from 4 g/d of IPE used in a Western population (183 μg/mL) [106].

In conclusion, Mechanistic and clinical studies support that LC ω-3 PUFAs have protective effects on the CV system via multiple pathways. LC ω-3 PUFAs lower TG concentrations, ameliorate inflammation, directly act on blood vessels, and improve vascular endothelial cell function, including vasodilation. In addition, LC ω-3 PUFAs act on platelets to reduce abnormal activation and serve as a component of the blood vessel wall to have a vascular protective effect. Although DHA has shown a neuroprotective effect, evidence of the CV benefits is limited. By contrast, as reported in JELIS [87] and REDUCE-IT [5], long-term administration of EPA alone was effective in reducing CV events, which was not found in STRENGTH, which used EPA and DHA in combination [6]. For these reasons, a potential strategy to be considered in designing future clinical trials would be to use EPA alone or to combine EPA and DHA for therapeutic regimens of LC ω-3 PUFAs. In addition, further studies are warranted to investigate the similarities and differences in the underlying mechanisms linked with specific effects of EPA and DHA.

## Acknowledgments

The authors’ responsibilities were as follows—JHB, HL, and SL: responsible for the study concept, design, and literature search; JHB: performed the analysis; JHB, HL, and SL: conducted interpretation of data and critical revision of the study material; JHB, HL, and SL: contributed to the drafting of the article; SL: supervised the study; and all authors: read and approved the final manuscript.

### Author disclosures

The authors report no conflicts of interest.

### Funding

The authors reported no funding received for this study.

## Appendix A Supplementary data

Multimedia component1 Multimedia component1

## References

[1] Ross A.C., Credo R. (2014).

[2] Siscovick D.S., Barringer T.A., Fretts A.M., Wu J.H., Lichtenstein A.H., Costello R.B. (2017). Omega-3 polyunsaturated fatty acid (fish oil) supplementation and the prevention of clinical cardiovascular disease: a science advisory from the American Heart Association. Circulation.

[3] Rimm E.B., Appel L.J., Chiuve S.E., Djoussé L., Engler M.B., Kris-Etherton P.M. (2018). Seafood long-chain n-3 polyunsaturated fatty acids and cardiovascular disease: a science advisory from the American Heart Association. Circulation.

[4] Harris W.S., Tintle N.L., Imamura F., Qian F., Korat A.V.A., Marklund M. (2021). Blood n-3 fatty acid levels and total and cause-specific mortality from 17 prospective studies. Nat. Commun..

[5] Bhatt D.L., Steg P.G., Miller M., Brinton E.A., Jacobson T.A., Ketchum S.B. (2019). Cardiovascular risk reduction with icosapent ethyl for hypertriglyceridemia. N. Engl. J. Med..

[6] Nicholls S.J., Lincoff A.M., Garcia M., Bash D., Ballantyne C.M., Barter P.J. (2020). Effect of high-dose omega-3 fatty acids vs corn oil on major adverse cardiovascular events in patients at high cardiovascular risk: the STRENGTH randomized clinical trial. JAMA.

[7] Lands B. (2022). Lipid nutrition: “in silico” studies and undeveloped experiments. Prog. Lipid Res..

[8] (2010). Fats and fatty acids in human nutrition. Report of an expert consultation. FAO Food Nutr. Pap..

[9] Clark C., Lands B. (2015). Creating benefits from omega-3 functional foods and nutraceuticals. Food Nutr. Sci..

[10] Hibbeln J.R., Nieminen L.R., Blasbalg T.L., Riggs J.A., Lands W.E. (2006). Healthy intakes of n-3 and n-6 fatty acids: estimations considering worldwide diversity. Am. J. Clin. Nutr..

[11] Wood K.E., Mantzioris E., Gibson R.A., Ramsden C.E., Muhlhausler B.S. (2015). The effect of modifying dietary LA and ALA intakes on omega-3 long chain polyunsaturated fatty acid (n-3 LCPUFA) status in human adults: a systematic review and commentary. Prostaglandins Leukot. Essent. Fatty Acids..

[12] Valentine R.C., Valentine D.L. (2004). Omega-3 fatty acids in cellular membranes: a unified concept. Prog. Lipid Res..

[13] Borow K.M., Nelson J.R., Mason R.P. (2015). Biologic plausibility, cellular effects, and molecular mechanisms of eicosapentaenoic acid (EPA) in atherosclerosis. Atherosclerosis.

[14] Burdge G.C., Finnegan Y.E., Minihane A.M., Williams C.M., Wootton S.A. (2003). Effect of altered dietary n-3 fatty acid intake upon plasma lipid fatty acid composition, conversion of [13C]alpha-linolenic acid to longer-chain fatty acids and partitioning towards beta-oxidation in older men. Br. J. Nutr..

[15] Baker E.J., Miles E.A., Burdge G.C., Yaqoob P., Calder P.C. (2016). Metabolism and functional effects of plant-derived omega-3 fatty acids in humans. Prog. Lipid Res..

[16] Sioen I., van Lieshout L., Eilander A., Fleith M., Lohner S., Szommer A. (2017). Systematic review on N-3 and N-6 polyunsaturated fatty acid intake in European countries in light of the current recommendations–focus on specific population groups. Ann. Nutr. Metab..

[17] Betz J.M., Blackman M.R., Coates P.M., Cragg G.M., Levine M., Moss J. (2013).

[18] Whelan J., Jahns L., Kavanagh K. (2009). Docosahexaenoic acid: measurements in food and dietary exposure. Prostaglandins Leukot. Essent. Fatty Acids..

[19] Saini R.K., Keum Y.S. (2018). Omega-3 and omega-6 polyunsaturated fatty acids: dietary sources, metabolism, and significance - a review. Life Sci.

[20] Hibbeln J.R., Spiller P., Brenna J.T., Golding J., Holub B.J., Harris W.S. (2019). Relationships between seafood consumption during pregnancy and childhood and neurocognitive development: two systematic reviews. Prostaglandins Leukot. Essent. Fatty Acids..

[21] Mason R.P., Libby P., Bhatt D.L. (2020). Emerging mechanisms of cardiovascular protection for the omega-3 fatty acid eicosapentaenoic acid. Arterioscler. Thromb. Vasc. Biol..

[22] Serhan C.N., Chiang N., Van Dyke T.E. (2008). Resolving inflammation: dual anti-inflammatory and pro-resolution lipid mediators. Nat. Rev. Immunol..

[23] Calder P.C. (2016). Docosahexaenoic acid. Ann. Nutr. Metab..

[24] O’Keefe E.L., Harris W.S., DiNicolantonio J.J., Elagizi A., Milani R.V., Lavie C.J. (2019). Sea change for marine omega-3s: randomized trials show fish oil reduces cardiovascular events. Mayo Clin. Proc..

[25] Lavie C.J., Milani R.V., Mehra M.R., Ventura H.O. (2009). Omega-3 polyunsaturated fatty acids and cardiovascular diseases. J. Am. Coll. Cardiol..

[26] EFSA Panel on Dietetic Products, Nutrition, and Allergies (NDA) (2010). Scientific opinion on dietary reference values for fats, including saturated fatty acids, polyunsaturated fatty acids, monounsaturated fatty acids, trans fatty acids, and cholesterol. EFSA J.

[27] Borén J., Taskinen M.R., Björnson E., Packard C.J. (2022). Metabolism of triglyceride-rich lipoproteins in health and dyslipidaemia. Nat. Rev. Cardiol..

[28] Dow C., Mangin M., Balkau B., Affret A., Boutron-Ruault M.C., Clavel-Chapelon F. (2016). Fatty acid consumption and incident type 2 diabetes: an 18-year follow-up in the female E3N (Etude Epidémiologique aupres des femmes de la Mutuelle Générale de l’Education Nationale) prospective cohort study. Br. J. Nutr..

[29] Bowman L., Mafham M., Wallendszus K., Stevens W., Buck G., ASCEND Study Collaborative Group (2018). Effects of n-3 fatty acid supplements in diabetes mellitus. N. Engl. J. Med..

[30] Zirpoli H., Chang C.L., Carpentier Y.A., Michael-Titus A.T., Ten V.S., Deckelbaum R.J. (2020). Novel approaches for omega-3 fatty acid therapeutics: chronic versus acute administration to protect heart, brain, and spinal cord. Annu. Rev. Nutr..

[31] Kromhout D., Yasuda S., Geleijnse J.M., Shimokawa H. (2012). Fish oil and omega-3 fatty acids in cardiovascular disease: do they really work?. Eur. Heart J..

[32] Björkegren J.L.M., Lusis A.J. (2022). Atherosclerosis: recent developments. Cell.

[33] Chang C.L., Deckelbaum R.J. (2013). Omega-3 fatty acids: mechanisms underlying ‘protective effects’ in atherosclerosis. Curr. Opin. Lipidol..

[34] Serhan C.N., Clish C.B., Brannon J., Colgan S.P., Chiang N., Gronert K. (2000). Novel functional sets of lipid-derived mediators with antiinflammatory actions generated from omega-3 fatty acids via cyclooxygenase 2-nonsteroidal antiinflammatory drugs and transcellular processing. J. Exp. Med..

[35] Norris P.C., Skulas-Ray A.C., Riley I., Richter C.K., Kris-Etherton P.M., Jensen G.L. (2018). Identification of specialized pro-resolving mediator clusters from healthy adults after intravenous low-dose endotoxin and omega-3 supplementation: a methodological validation. Sci. Rep..

[36] Spite M., Clària J., Serhan C.N. (2014). Resolvins, specialized proresolving lipid mediators, and their potential roles in metabolic diseases. Cell Metab.

[37] Akagi D., Chen M., Toy R., Chatterjee A., Conte M.S. (2015). Systemic delivery of proresolving lipid mediators resolvin D2 and maresin 1 attenuates intimal hyperplasia in mice. FASEB J.

[38] Makino Y., Miyahara T., Nitta J., Miyahara K., Seo A., Kimura M. (2019). Proresolving lipid mediators resolvin D1 and protectin D1 isomer attenuate neointimal hyperplasia in the rat carotid artery balloon injury model. J. Surg. Res..

[39] Kain V., Ingle K.A., Colas R.A., Dalli J., Prabhu S.D., Serhan C.N. (2015). Resolvin D1 activates the inflammation resolving response at splenic and ventricular site following myocardial infarction leading to improved ventricular function. J. Mol. Cell. Cardiol..

[40] Sugimoto S., Mena H.A., Sansbury B.E., Kobayashi S., Tsuji T., Wang C.H. (2022). Brown adipose tissue-derived MaR2 contributes to cold-induced resolution of inflammation. Nat. Metab..

[41] Serhan C.N. (2014). Pro-resolving lipid mediators are leads for resolution physiology. Nature.

[42] Harris W.S., Ginsberg H.N., Arunakul N., Shachter N.S., Windsor S.L., Adams M. (1997). Safety and efficacy of Omacor in severe hypertriglyceridemia. J. Cardiovasc. Risk..

[43] Pownall H.J., Brauchi D., Kilinç C., Osmundsen K., Pao Q., Payton-Ross C. (1999). Correlation of serum triglyceride and its reduction by omega-3 fatty acids with lipid transfer activity and the neutral lipid compositions of high-density and low-density lipoproteins. Atherosclerosis.

[44] Kastelein J.J., Maki K.C., Susekov A., Ezhov M., Nordestgaard B.G., Machielse B.N. (2014). Omega-3 free fatty acids for the treatment of severe hypertriglyceridemia: the EpanoVa for lowering very high triglycerides (EVOLVE) trial. J. Clin. Lipidol..

[45] Bays H.E., Ballantyne C.M., Kastelein J.J., Isaacsohn J.L., Braeckman R.A., Soni P.N. (2011). Eicosapentaenoic acid ethyl ester (AMR101) therapy in patients with very high triglyceride levels (from the multi-center, placebo-controlled, randomized, double-blind, 12-week study with an open-label extension [MARINE] trial). Am. J. Cardiol..

[46] Davidson M.H., Stein E.A., Bays H.E., Maki K.C., Doyle R.T., Shalwitz R.A. (2007). Efficacy and tolerability of adding prescription omega-3 fatty acids 4 g/d to simvastatin 40 mg/d in hypertriglyceridemic patients: an 8-week, randomized, double-blind, placebo-controlled study. Clin. Ther..

[47] Maki K.C., Orloff D.G., Nicholls S.J., Dunbar R.L., Roth E.M., Curcio D. (2013). A highly bioavailable omega-3 free fatty acid formulation improves the cardiovascular risk profile in high-risk, statin-treated patients with residual hypertriglyceridemia (the ESPRIT trial). Clin. Ther..

[48] Ballantyne C.M., Bays H.E., Kastelein J.J., Stein E., Isaacsohn J.L., Braeckman R.A. (2012). Efficacy and safety of eicosapentaenoic acid ethyl ester (AMR101) therapy in statin-treated patients with persistent high triglycerides (from the ANCHOR study). Am. J. Cardiol..

[49] Mori T.A., Burke V., Puddey I.B., Watts G.F., O’Neal D.N., Best J.D. (2000). Purified eicosapentaenoic and docosahexaenoic acids have differential effects on serum lipids and lipoproteins, LDL particle size, glucose, and insulin in mildly hyperlipidemic men. Am. J. Clin. Nutr..

[50] Rosenson R.S., Shaik A., Song W. (2021). New therapies for lowering triglyceride-rich lipoproteins: JACC Focus Seminar 3/4. J. Am. Coll. Cardiol..

[51] Tanaka N., Irino Y., Shinohara M., Tsuda S., Mori T., Nagao M. (2018). Eicosapentaenoic acid-enriched high-density lipoproteins exhibit anti-atherogenic properties. Circ. J..

[52] Cho K.I., Yu J., Hayashi T., Han S.H., Koh K.K. (2019). Strategies to overcome residual risk during statins era. Circ. J..

[53] Allaire J., Couture P., Leclerc M., Charest A., Marin J., Lépine M.C. (2016). A randomized, crossover, head-to-head comparison of eicosapentaenoic acid and docosahexaenoic acid supplementation to reduce inflammation markers in men and women: the Comparing EPA to DHA (ComparED) Study. Am. J. Clin. Nutr..

[54] Zaloga G.P. (2021). Narrative review of n-3 polyunsaturated fatty acid supplementation upon immune functions, resolution molecules and lipid peroxidation. Nutrients.

[55] Liu G., Liu Q., Shen Y., Kong D., Gong Y., Tao B. (2018). Early treatment with resolvin E1 facilitates myocardial recovery from ischaemia in mice. Br. J. Pharmacol..

[56] Bazan N.G. (2005). Neuroprotectin D1 (NPD1): a DHA-derived mediator that protects brain and retina against cell injury-induced oxidative stress. Brain Pathol.

[57] Zuo G., Zhang D., Mu R., Shen H., Li X., Wang Z. (2018). Resolvin D2 protects against cerebral ischemia/reperfusion injury in rats. Mol. Brain..

[58] Hosogoe N., Ishikawa S., Yokoyama N., Kozuma K., Isshiki T. (2017). Add-on antiplatelet effects of eicosapentaenoic acid with tailored dose setting in patients on dual antiplatelet therapy. Int. Heart J..

[59] Knapp H.R., Reilly I.A., Alessandrini P., FitzGerald G.A. (1986). In vivo indexes of platelet and vascular function during fish-oil administration in patients with atherosclerosis. N. Engl. J. Med..

[60] von Schacky C., Fischer S., Weber P.C. (1985). Long-term effects of dietary marine omega-3 fatty acids upon plasma and cellular lipids, platelet function, and eicosanoid formation in humans. J. Clin. Invest..

[61] Moertl D., Berger R., Hammer A., Hutuleac R., Koppensteiner R., Kopp C.W. (2011). Dose-dependent decrease of platelet activation and tissue factor by omega-3 polyunsaturated fatty acids in patients with advanced chronic heart failure. Thromb. Haemost..

[62] Moertl D., Hammer A., Steiner S., Hutuleac R., Vonbank K., Berger R. (2011). Dose-dependent effects of omega-3-polyunsaturated fatty acids on systolic left ventricular function, endothelial function, and markers of inflammation in chronic heart failure of nonischemic origin: a double-blind, placebo-controlled, 3-arm study. Am. Heart J..

[63] Woodman R.J., Mori T.A., Burke V., Puddey I.B., Barden A., Watts G.F. (2003). Effects of purified eicosapentaenoic acid and docosahexaenoic acid on platelet, fibrinolytic and vascular function in hypertensive type 2 diabetic patients. Atherosclerosis.

[64] Phang M., Sinclair A.J., Lincz L.F., Garg M.L. (2012). Gender-specific inhibition of platelet aggregation following omega-3 fatty acid supplementation. Nutr. Metab. Cardiovasc. Dis..

[65] Backes J., Anzalone D., Hilleman D., Catini J. (2016). The clinical relevance of omega-3 fatty acids in the management of hypertriglyceridemia. Lipids Health Dis.

[66] O’Mahoney L.L., Matu J., Price O.J., Birch K.M., Ajjan R.A., Farrar D. (2018). Omega-3 polyunsaturated fatty acids favourably modulate cardiometabolic biomarkers in type 2 diabetes: a meta-analysis and meta-regression of randomized controlled trials. Cardiovasc. Diabetol..

[67] Smith G.I., Julliand S., Reeds D.N., Sinacore D.R., Klein S., Mittendorfer B. (2015). Fish oil-derived n-3 PUFA therapy increases muscle mass and function in healthy older adults. Am. J. Clin. Nutr..

[68] Al Rijjal D., Liu Y., Lai M., Song Y., Danaei Z., Wu A. (2021). Vascepa protects against high-fat diet-induced glucose intolerance, insulin resistance, and impaired beta-cell function. iScience.

[69] Zhou Y., Tian C., Jia C. (2012). Association of fish and n-3 fatty acid intake with the risk of type 2 diabetes: a meta-analysis of prospective studies. Br. J. Nutr..

[70] Wallin A., Di Giuseppe D., Orsini N., Patel P.S., Forouhi N.G., Wolk A. (2012). Fish consumption, dietary long-chain n-3 fatty acids, and risk of type 2 diabetes: systematic review and meta-analysis of prospective studies. Diabetes Care.

[71] Brown T.J., Brainard J., Song F., Wang X., Abdelhamid A., Hooper L. (2019). Omega-3, omega-6, and total dietary polyunsaturated fat for prevention and treatment of type 2 diabetes mellitus: systematic review and meta-analysis of randomised controlled trials. BMJ.

[72] Zhuang P., Li H., Jia W., Shou Q., Zhu Y., Mao L. (2021). Eicosapentaenoic and docosahexaenoic acids attenuate hyperglycemia through the microbiome-gut-organs axis in db/db mice. Microbiome.

[73] Menni C., Zierer J., Pallister T., Jackson M.A., Long T., Mohney R.P. (2017). Omega-3 fatty acids correlate with gut microbiome diversity and production of N-carbamylglutamate in middle aged and elderly women. Sci. Rep..

[74] Balfegó M., Canivell S., Hanzu F.A., Sala-Vila A., Martínez-Medina M., Murillo S. (2016). Effects of sardine-enriched diet on metabolic control, inflammation and gut microbiota in drug-naive patients with type 2 diabetes: a pilot randomized trial. Lipids Health Dis.

[75] Watson H., Mitra S., Croden F.C., Taylor M., Wood H.M., Perry S.L. (2018). A randomised trial of the effect of omega-3 polyunsaturated fatty acid supplements on the human intestinal microbiota. Gut.

[76] Caesar R., Tremaroli V., Kovatcheva-Datchary P., Cani P.D., Bäckhed F. (2015). Crosstalk between gut microbiota and dietary lipids aggravates WAT inflammation through TLR signaling. Cell Metab.

[77] Wu G.D., Chen J., Hoffmann C., Bittinger K., Chen Y.Y., Keilbaugh S.A. (2011). Linking long-term dietary patterns with gut microbial enterotypes. Science.

[78] Eslam M., Newsome P.N., Sarin S.K., Anstee Q.M., Targher G., Romero-Gomez M. (2020). A new definition for metabolic dysfunction-associated fatty liver disease: an international expert consensus statement. J. Hepatol..

[79] Lim S., Kim J.W., Targher G. (2021). Links between metabolic syndrome and metabolic dysfunction-associated fatty liver disease. Trends Endocrinol. Metab..

[80] (2016). European Association for the Study of the Liver (EASL), European Association for the Study of Diabetes (EASD), European Association for the Study of Obesity (EASO), EASL-EASD-EASO Clinical Practice Guidelines for the management of non-alcoholic fatty liver disease. J. Hepatol..

[81] Chalasani N., Younossi Z., Lavine J.E., Diehl A.M., Brunt E.M., Cusi K. (2012). The diagnosis and management of non-alcoholic fatty liver disease: practice guideline by the American Association for the Study of Liver Diseases, American College of Gastroenterology, and the American Gastroenterological Association. Hepatology.

[82] Lepretti M., Martucciello S., Burgos Aceves M.A., Putti R., Lionetti L. (2018). Omega-3 fatty acids and insulin resistance: focus on the regulation of mitochondria and endoplasmic reticulum stress. Nutrients.

[83] Pahlavani M., Ramalho T., Koboziev I., LeMieux M.J., Jayarathne S., Ramalingam L. (2017). Adipose tissue inflammation in insulin resistance: review of mechanisms mediating anti-inflammatory effects of omega-3 polyunsaturated fatty acids. J. Investig. Med..

[84] de Castro G.S., Calder P.C. (2018). Non-alcoholic fatty liver disease and its treatment with n-3 polyunsaturated fatty acids. Clin. Nutr..

[85] Zhang B., Xiong K., Cai J., Ma A. (2020). Fish consumption and coronary heart disease: a meta-analysis. Nutrients.

[86] (1999). Dietary supplementation with n-3 polyunsaturated fatty acids and vitamin E after myocardial infarction: results of the GISSI-Prevenzione trial. Gruppo Italiano per lo Studio della Sopravvivenza nell’Infarto miocardico. Lancet.

[87] Yokoyama M., Origasa H., Matsuzaki M., Matsuzawa Y., Saito Y., Ishikawa Y. (2007). Effects of eicosapentaenoic acid on major coronary events in hypercholesterolaemic patients (JELIS): a randomised open-label, blinded endpoint analysis. Lancet.

[88] Manson J.E., Cook N.R., Lee I.M., Christen W., Bassuk S.S., Mora S. (2019). Marine n-3 fatty acids and prevention of cardiovascular disease and cancer. N. Engl. J. Med..

[89] Doi T., Langsted A., Nordestgaard B.G. (2021). A possible explanation for the contrasting results of REDUCE-IT vs. STRENGTH: cohort study mimicking trial designs. Eur. Heart J..

[90] Budoff M.J., Bhatt D.L., Kinninger A., Lakshmanan S., Muhlestein J.B., Le V.T. (2020). Effect of icosapent ethyl on progression of coronary atherosclerosis in patients with elevated triglycerides on statin therapy: final results of the EVAPORATE trial. Eur. Heart J..

[91] Eritsland J., Arnesen H., Grønseth K., Fjeld N.B., Abdelnoor M. (1996). Effect of dietary supplementation with n-3 fatty acids on coronary artery bypass graft patency. Am. J. Cardiol..

[92] Albert C.M., Cook N.R., Pester J., Moorthy M.V., Ridge C., Danik J.S. (2021). Effect of marine omega-3 fatty acid and vitamin D supplementation on incident atrial fibrillation: a randomized clinical trial. JAMA.

[93] Roncaglioni M.C., Tombesi M., Avanzini F., Barlera S., Caimi V., Risk and Prevention Study Collaborative Group (2013). n-3 fatty acids in patients with multiple cardiovascular risk factors. N. Engl. J. Med..

[94] Gencer B., Djousse L., Al-Ramady O.T., Cook N.R., Manson J.E., Albert C.M. (2021). Effect of long-term marine ɷ-3 fatty acids supplementation on the risk of atrial fibrillation in randomized controlled trials of cardiovascular outcomes: a systematic review and meta-analysis. Circulation.

[95] Kalstad A.A., Myhre P.L., Laake K., Tveit S.H., Schmidt E.B., Smith P. (2021). Effects of n-3 fatty acid supplements in elderly patients after myocardial infarction: a randomized, controlled trial. Circulation.

[96] Fatkin D., Cox C.D., Martinac B. (2022). Fishing for links between omega-3 fatty acids and atrial fibrillation. Circulation.

[97] Jakob D., Klesen A., Allegrini B., Darkow E., Aria D., Emig R. (2021). Piezo1 and BKCa channels in human atrial fibroblasts: interplay and remodelling in atrial fibrillation. J. Mol. Cell. Cardiol..

[98] Romero L.O., Massey A.E., Mata-Daboin A.D., Sierra-Valdez F.J., Chauhan S.C., Cordero-Morales J.F. (2019). Dietary fatty acids fine-tune Piezo1 mechanical response. Nat. Commun..

[99] Bhatt D.L., Steg P.G., Miller M., Brinton E.A., Jacobson T.A., Jiao L. (2019). Reduction in first and total ischemic events with icosapent ethyl across baseline triglyceride tertiles. J. Am. Coll. Cardiol..

[100] Verma S., Bhatt D.L., Steg P.G., Miller M., Brinton E.A., Jacobson T.A. (2021). Icosapent ethyl reduces ischemic events in patients with a history of previous coronary artery bypass grafting: REDUCE-IT CABG. Circulation.

[101] Gaba P., Bhatt D.L., Steg P.G., Miller M., Brinton E.A., Jacobson T.A. (2022). Prevention of cardiovascular events and mortality with icosapent ethyl in patients with prior myocardial infarction. J. Am. Coll. Cardiol..

[102] Singh N., Bhatt D.L., Miller M., Steg P.G., Brinton E.A., Jacobson T.A. (2022). Consistency of benefit of icosapent ethyl by background statin type in REDUCE-IT. J. Am. Coll. Cardiol..

[103] Galan P., Kesse-Guyot E., Czernichow S., Briancon S., Blacher J., Hercberg S. (2010). Effects of B vitamins and omega 3 fatty acids on cardiovascular diseases: a randomised placebo controlled trial. BMJ.

[104] Skulas-Ray A.C., Wilson P.W.F., Harris W.S., Brinton E.A., Kris-Etherton P.M., Richter C.K. (2019). Omega-3 fatty acids for the management of hypertriglyceridemia: a science advisory from the American Heart Association. Circulation.

[105] Mach F., Baigent C., Catapano A.L., Koskinas K.C., Casula M., Badimon L. (2020). 2019 ESC/EAS Guidelines for the management of dyslipidaemias: lipid modification to reduce cardiovascular risk. Eur. Heart J..

[106] Bays H.E., Ballantyne C.M., Doyle R.T., Juliano R.A., Philip S. (2016). Icosapent ethyl: eicosapentaenoic acid concentration and triglyceride-lowering effects across clinical studies. Prostaglandins Other Lipid Mediat.

[107] Tavazzi L., Maggioni A.P., Marchioli R., Barlera S., Franzosi M.G., Latini R. (2008). Effect of n-3 polyunsaturated fatty acids in patients with chronic heart failure (the GISSI-HF trial): a randomised, double-blind, placebo-controlled trial. Lancet.

[108] Rauch B., Schiele R., Schneider S., Diller F., Victor N., Gohlke H. (2010). OMEGA, a randomized, placebo-controlled trial to test the effect of highly purified omega-3 fatty acids on top of modern guideline-adjusted therapy after myocardial infarction. Circulation.

[109] Bosch J., Gerstein H.C., Dagenais G.R., Díaz R., Dyal L., ORIGIN Trial Investigators (2012). n-3 fatty acids and cardiovascular outcomes in patients with dysglycemia. N. Engl. J. Med..

[110] Macchia A., Grancelli H., Varini S., Nul D., Laffaye N., Mariani J. (2013). Omega-3 fatty acids for the prevention of recurrent symptomatic atrial fibrillation: results of the FORWARD (randomized trial to assess efficacy of PUFA for the maintenance of sinus rhythm in persistent atrial fibrillation) trial. J. Am. Coll. Cardiol..

[111] Brouwer I.A., Zock P.L., Camm A.J., Böcker D., Hauer R.N., Wever E.F. (2006). Effect of fish oil on ventricular tachyarrhythmia and death in patients with implantable cardioverter defibrillators: the study on omega-3 fatty acids and ventricular arrhythmia (SOFA) randomized trial. JAMA.

[112] Kromhout D., Giltay E.J., Geleijnse J.M. (2010). Alpha Omega Trial Group, N-3 fatty acids and cardiovascular events after myocardial infarction. N. Engl. J. Med..

[113] Einvik G., Klemsdal T.O., Sandvik L., Hjerkinn E.M. (2010). A randomized clinical trial on n-3 polyunsaturated fatty acids supplementation and all-cause mortality in elderly men at high cardiovascular risk. Eur. J. Cardiovasc. Prev. Rehabil..

[114] Bonds D.E., Harrington M., Worrall B.B., Bertoni A.G., Eaton C.B., Writing Group for the AREDS2 Research Group (2014). Effect of long-chain ω-3 fatty acids and lutein + zeaxanthin supplements on cardiovascular outcomes: results of the Age-Related Eye Disease Study 2 (AREDS2) randomized clinical trial. JAMA Intern. Med..

