# Corrigendum to “The Potential Cardiometabolic Effects of Long-Chain ω-3 Polyunsaturated Fatty Acids: Recent Updates and Controversies” [Adv Nutr 14(4) (2023) 612–628]

**DOI:** 10.1016/j.advnut.2023.10.006

**Published:** 2023-10-26

**Authors:** Jae Hyun Bae, Hyunjung Lim, Soo Lim

**Affiliations:** 1 Department of Internal Medicine, Korea University Anam Hospital, Korea University College of Medicine, Seoul, Republic of Korea; 2 Department of Medical Nutrition, Research Institute of Medical Nutrition, Graduate School of East-West Medical Science, Kyung Hee University, Yongin, Republic of Korea; 3 Department of Internal Medicine, Seoul National University Bundang Hospital, Seoul National University College of Medicine, Seongnam, Republic of Korea

The authors would like to express their regrets and acknowledge that the FORWARD study, included in the meta-analysis, primarily aimed at assessing the efficacy of polyunsaturated fatty acids in preventing recurrent atrial fibrillation in patients with a normal sinus rhythm. Therefore, in the analysis results, the term “new-onset atrial fibrillation” should have read ‘new-onset or recurrent atrial fibrillation’. The authors sincerely apologize for any inconvenience this may have caused.

