# Supplementary material for: The Potential Cardiometabolic Effects of Long-Chain ω-3 Polyunsaturated Fatty Acids: Recent Updates and Controversies
Source: Adv Nutr. 2023 Apr 7;14(4):612–28. doi: 10.1016/j.advnut.2023.03.014 (PMC10334139; doi:10.1016/j.advnut.2023.03.014)
Supplement: Multimedia component1 [file mmc1.docx]

**Title: The potential cardiometabolic benefits of omega-3 polyunsaturated fatty acids focusing on recent updates and controversies**

**First Author:** Jae Hyun Bae, Hyunjung Lim

**Online Supplementary material**

**Supplemental Figure 1** Protective role of omega-3 fatty acids in the development of atherosclerosis. IL-6, interleukin-6; MCP-1, matrix metalloprotein-1; PDGF, platelet-derived growth factor; TNF-α, tumor necrosis factor-α.

**Supplemental Figure 2** The major role of specialized pro-resolving mediators produced from enzymatic oxygenation of omega-3 fatty acids and their potential benefits in the cardiovascular system.

**Supplemental Figure 3** Meta-analysis of the effects of long-chain omega-3 polyunsaturated fatty acid supplementation on three-point major adverse cardiovascular events by types, comparators, and prevention groups.

**Supplemental Figure 4** Dose-response association of EPA with three-point major adverse cardiovascular events.

**Supplemental Figure 5** Meta-analysis of the effects of long-chain omega-3 polyunsaturated fatty acid supplementation on all-cause mortality.

**Supplemental Figure 6** Meta-analysis of the effects of long-chain omega-3 polyunsaturated fatty acid supplementation on hospitalization for heart failure.

**Supplemental Figure 7** Meta-analysis of the effects of long-chain omega-3 polyunsaturated fatty acid supplementation on new-onset atrial fibrillation

**Supplemental Figure 8** Dose-response association of EPA with new-onset atrial fibrillation.

**Supplemental Figure 1** Protective role of omega-3 fatty acids in the development of atherosclerosis. IL-6, interleukin-6; MCP-1, matrix metalloprotein-1; PDGF, platelet-derived growth factor; TNF-α, tumor necrosis factor-α.


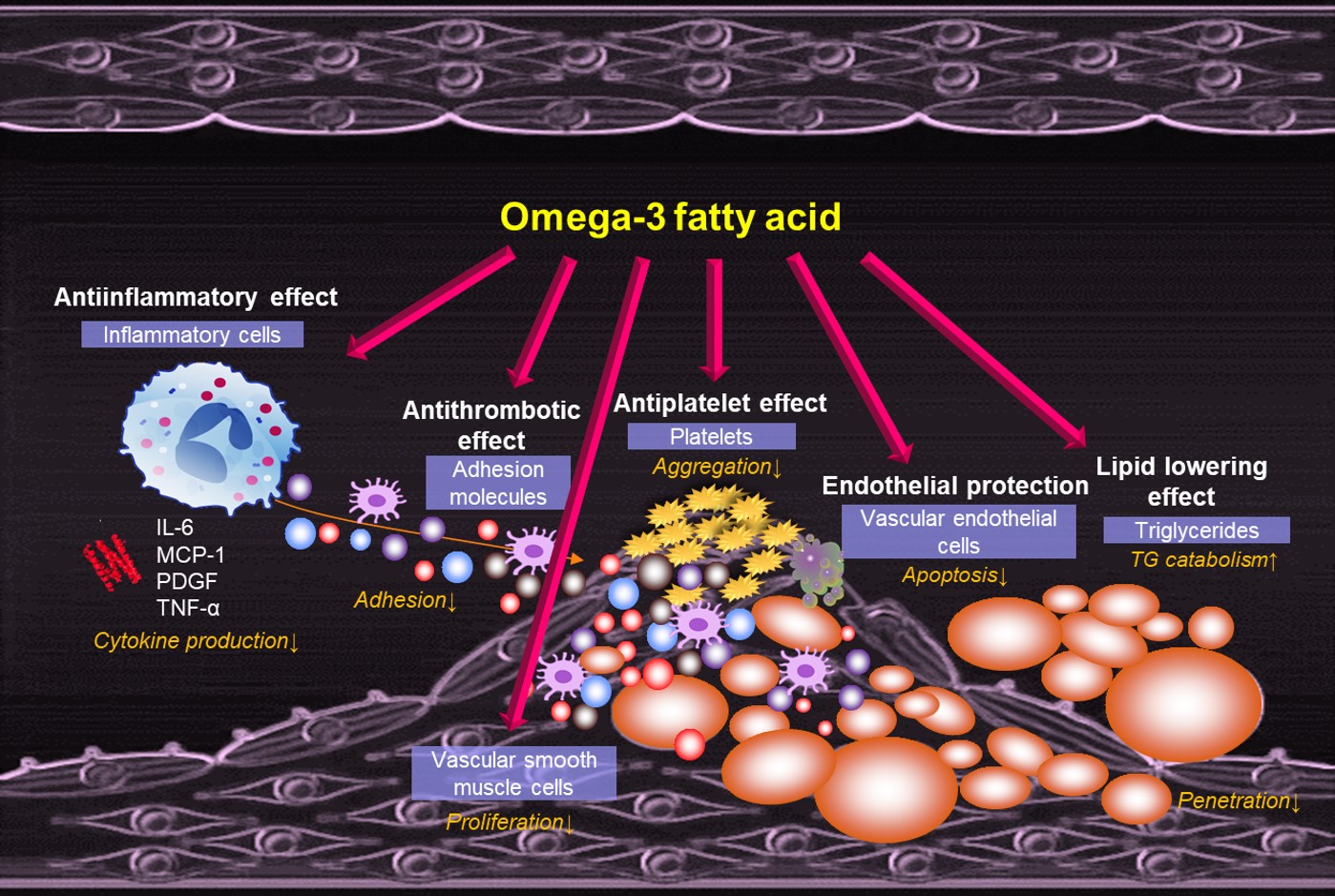


**Supplemental Figure 2** The major role of specialized pro-resolving mediators produced from enzymatic oxygenation of omega-3 fatty acids and their potential benefits in the cardiovascular system.


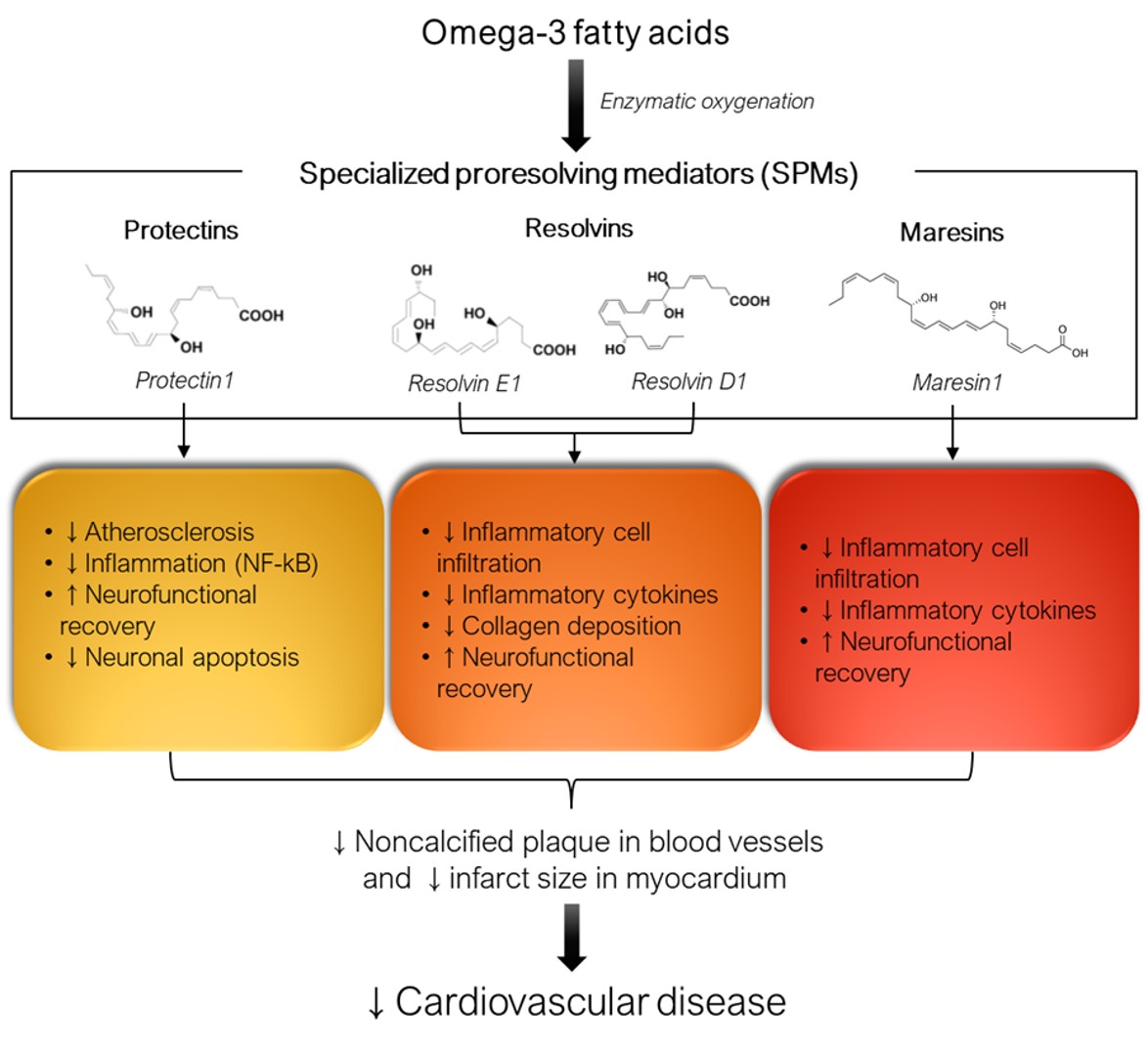


**Supplemental Figure 3** Meta-analysis of the effects of long-chain omega-3 polyunsaturated fatty acid supplementation on three-point major adverse cardiovascular events by types, comparators, and prevention groups.

**(A) Types of long-chain omega-3 polyunsaturated fatty acids**

**
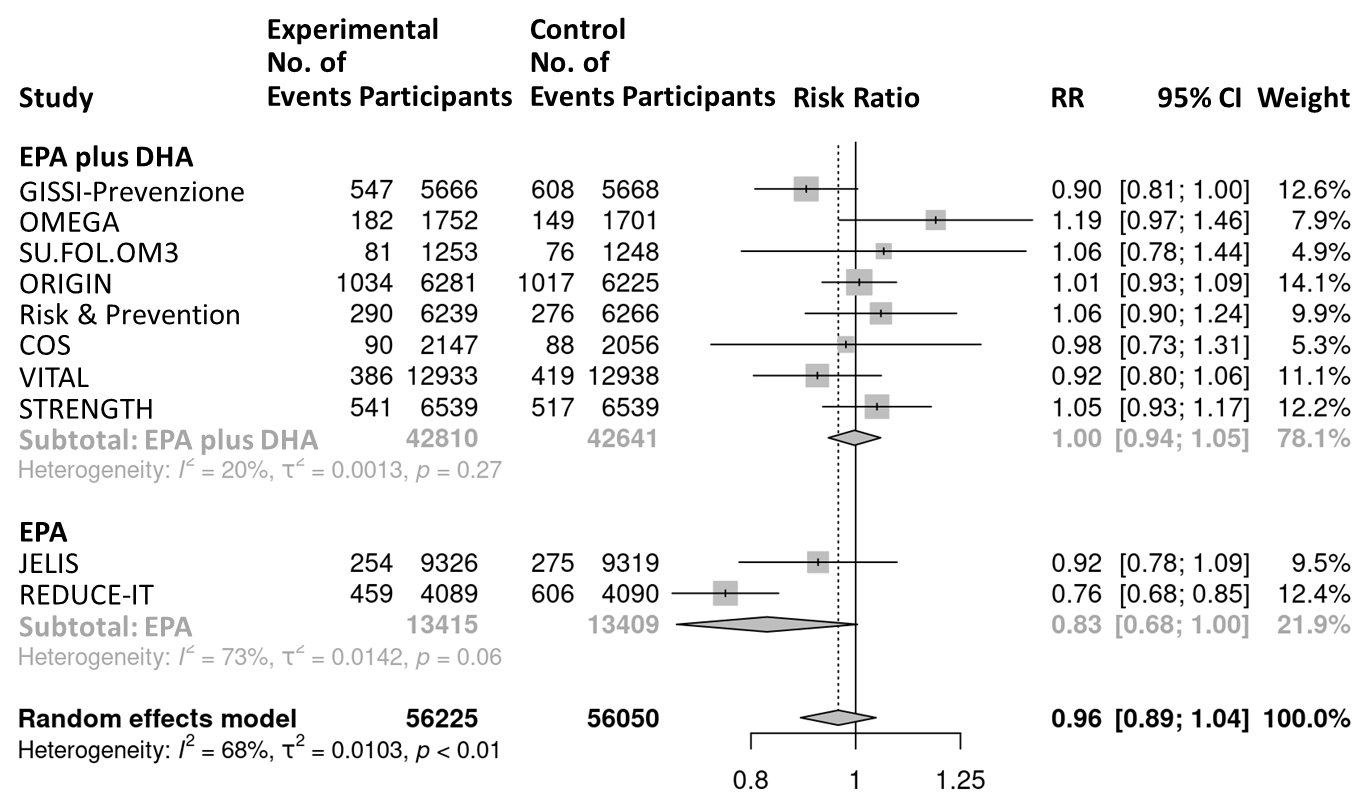
**

ASCEND, A Study of Cardiovascular Events iN Diabetes; CI, confidence interval; COS, CV Outcome Study; DM, diabetes mellitus; GISSI-Prevenzione, Gruppo Italiano per lo Studio della Sopravvivenza nell’Infarto miocardico-Prevenzione; JELIS, Japan EPA Lipid Intervention Study; MI, myocardial infarction; ORIGIN, Outcome Reduction with an Initial Glargine Intervention; REDUCE-IT, Reduction of Cardiovascular Events with Icosapent Ethyl-Intervention Trial; STRENGTH, Long-Term Outcome Study to Assess STatin Residual Risk Reduction with EpaNova in HiGh Cardiovascular risk PatienTs with Hypertriglyceridemia; SU.FOL.OM3, SUpplémentation en FOLates et OMega-3; VF, ventricular fibrillation; VITAL, VITamin D and OmegA-3 TriaL.

**(B) Comparators**

**
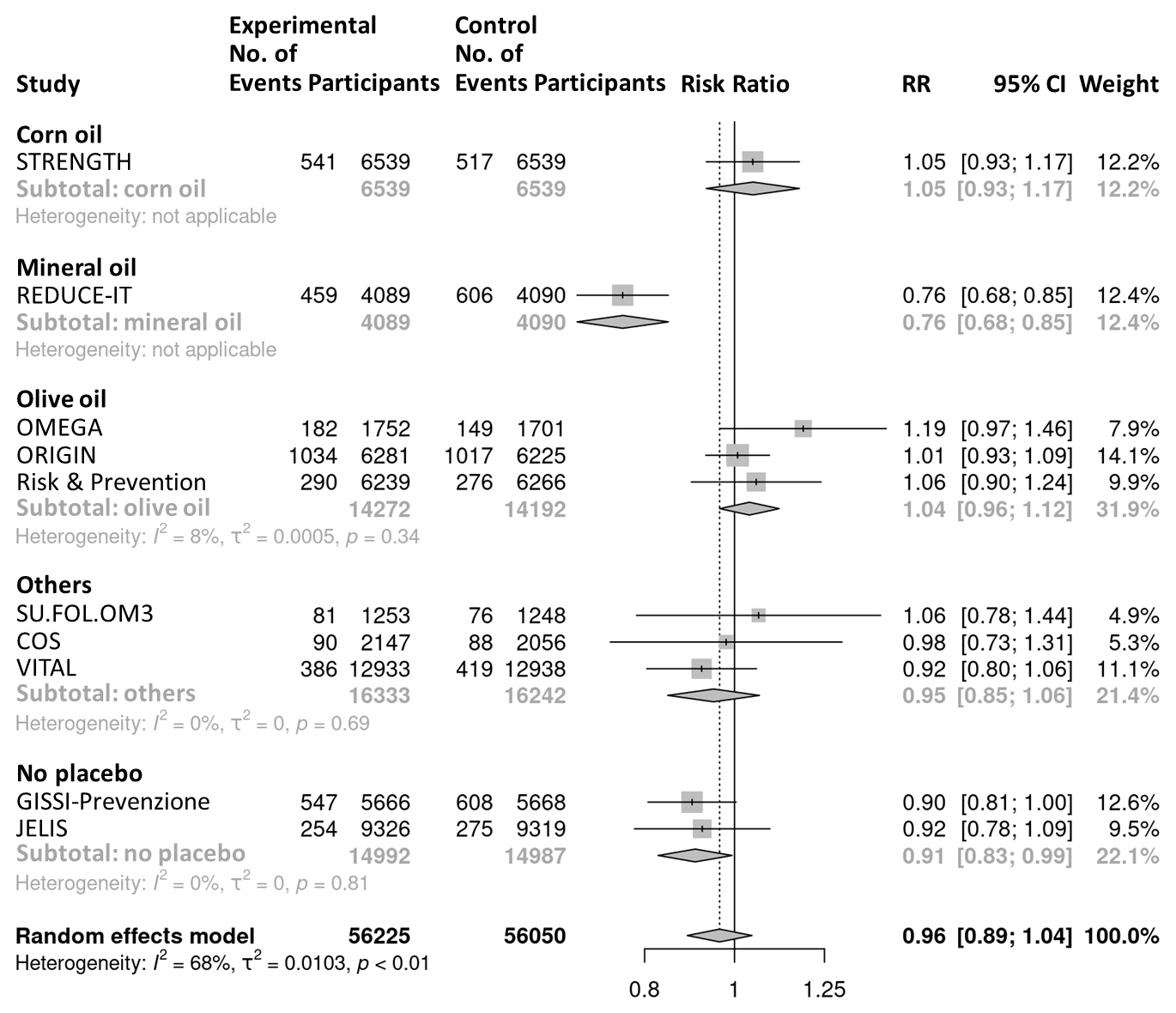
**

**(C) Prevention groups**

**
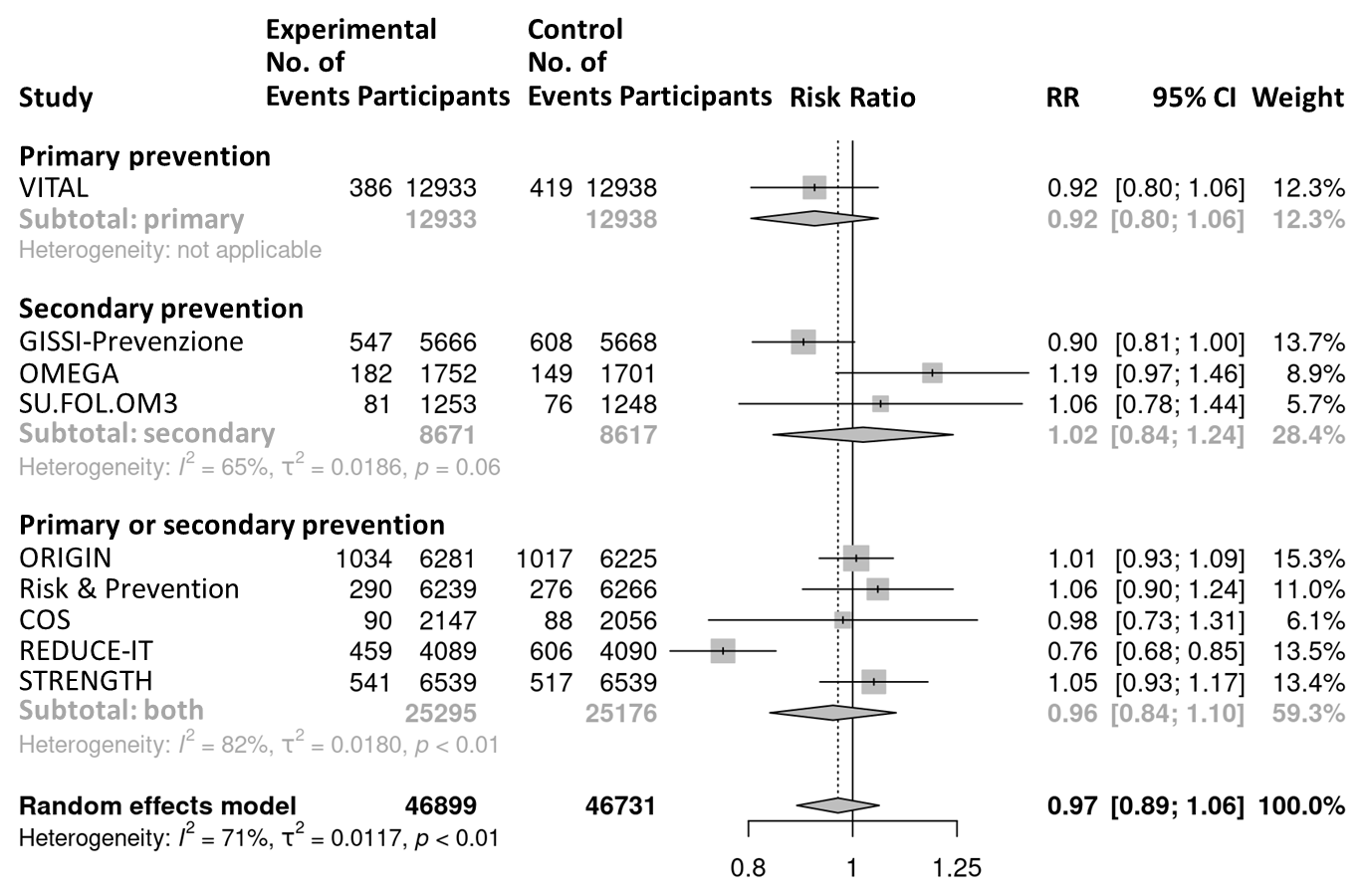
**

**Supplemental Figure 4** Dose-response association of EPA with three-point major adverse cardiovascular events.


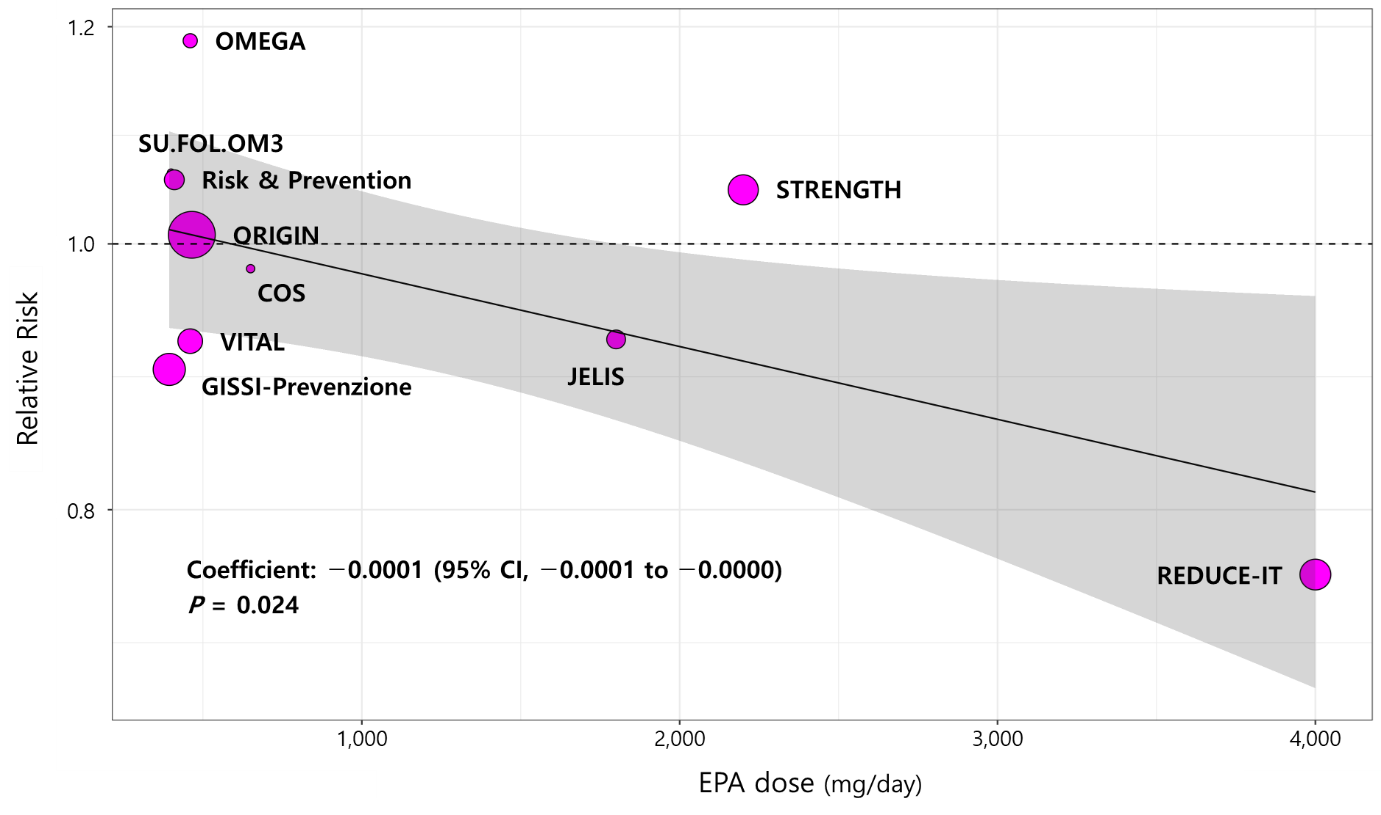


CI, confidence interval; COS, CV Outcome Study; EPA, eicosapentaenoic acid; GISSI-Prevenzione, Gruppo Italiano per lo Studio della Sopravvivenza nell’Infarto miocardico-Prevenzione; JELIS, Japan EPA Lipid Intervention Study; ORIGIN, Outcome Reduction with an Initial Glargine Intervention; REDUCE-IT, Reduction of Cardiovascular Events with Icosapent Ethyl-Intervention Trial; STRENGTH, Long-Term Outcome Study to Assess STatin Residual Risk Reduction with EpaNova in HiGh Cardiovascular risk PatienTs with Hypertriglyceridemia; SU.FOL.OM3, SUpplémentation en FOLates et OMega-3; VITAL, VITamin D and OmegA-3 TriaL.

**Supplemental Figure 5** Meta-analysis of the effects of long-chain omega-3 polyunsaturated fatty acid supplementation on all-cause mortality.


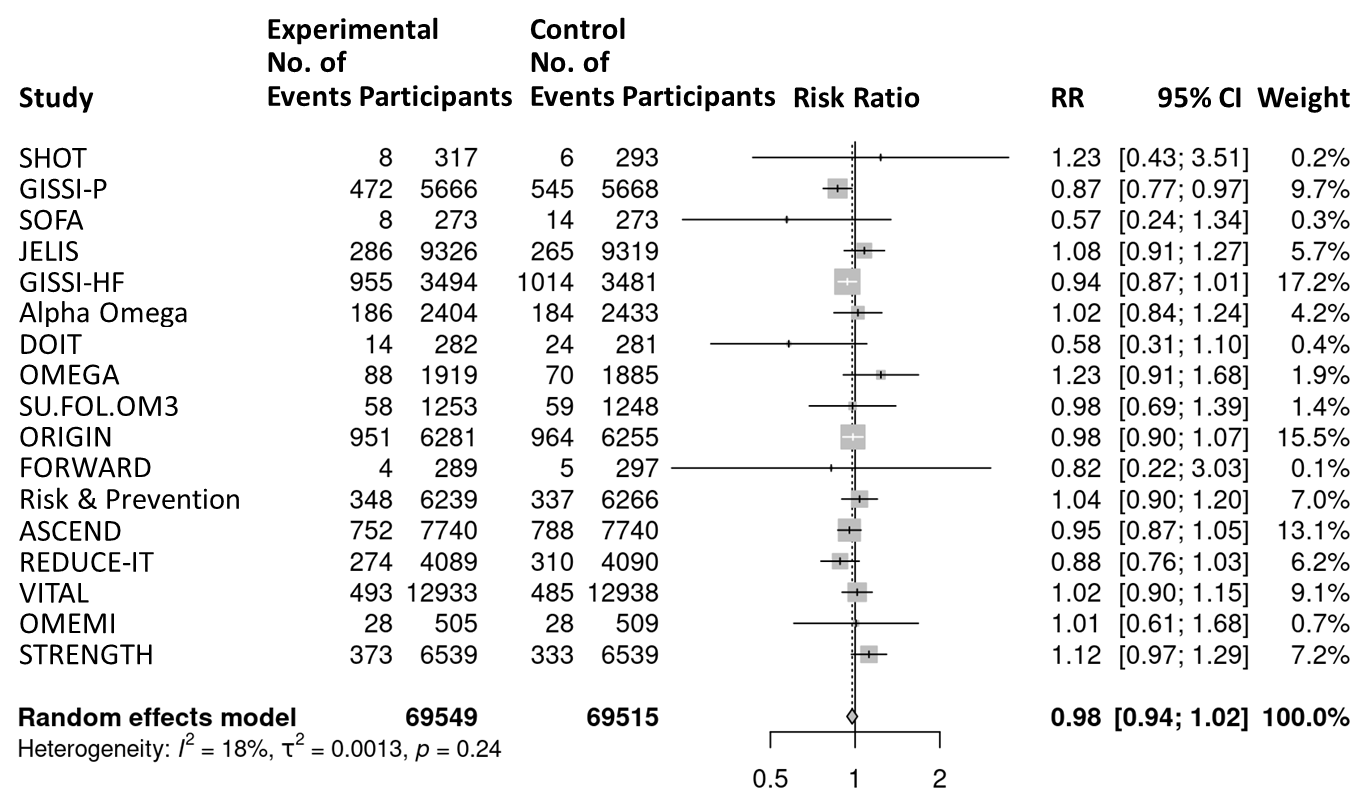


ASCEND, A Study of Cardiovascular Events iN Diabetes; DOIT, Diet and Omega-3 Intervention Trial; FORWARD, Fish Oil Research with ω-3 for Atrial Fibrillation Recurrence Delaying; GISSI-HF, Gruppo Italiano per lo Studio della Sopravvivenza nell’Infarto miocardico-Heart Failure; GISSI-Prevenzione, Gruppo Italiano per lo Studio della Sopravvivenza nell’Infarto miocardico-Prevenzione; JELIS, Japan EPA Lipid Intervention Study; OMEMI, OMega-3 fatty acids in Elderly patients with Myocardial Infarction; ORIGIN, Outcome Reduction with an Initial Glargine Intervention; REDUCE-IT, Reduction of Cardiovascular Events with Icosapent Ethyl-Intervention Trial; SHOT, SHunt Occlusion Trial; SOFA, Study on Omega-3 Fatty acids and ventricular Arrhythmia; STRENGTH, Long-Term Outcome Study to Assess STatin Residual Risk Reduction with EpaNova in HiGh Cardiovascular risk PatienTs with Hypertriglyceridemia; SU.FOL.OM3, SUpplémentation en FOLates et OMega-3; VITAL, VITamin D and OmegA-3 TriaL.

**Supplemental Figure 6** Meta-analysis of the effects of long-chain omega-3 polyunsaturated fatty acid supplementation on hospitalization for heart failure.


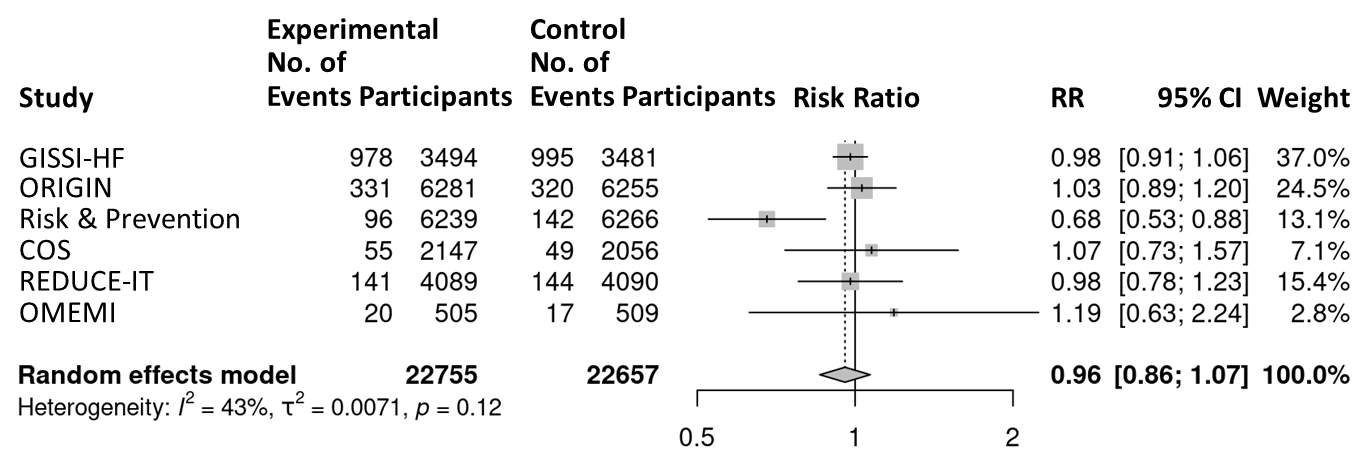


**Supplemental Figure 7** Meta-analysis of the effects of long-chain omega-3 polyunsaturated fatty acid supplementation on new-onset atrial fibrillation.


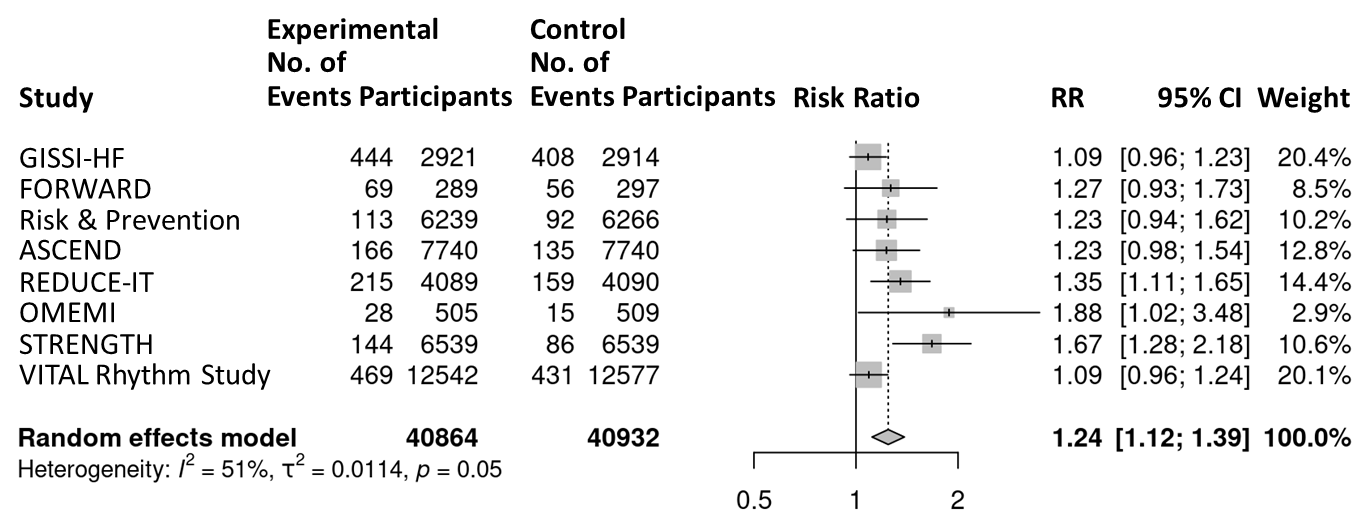


ASCEND, A Study of Cardiovascular Events iN Diabetes; COS, CV Outcome Study; DM, diabetes mellitus; DOIT, Diet and Omega-3 Intervention Trial; FORWARD, Fish Oil Research with ω-3 for Atrial Fibrillation Recurrence Delaying; GISSI-HF, Gruppo Italiano per lo Studio della Sopravvivenza nell’Infarto miocardico-Heart Failure; OMEMI, OMega-3 fatty acids in Elderly patients with Myocardial Infarction; ORIGIN, Outcome Reduction with an Initial Glargine Intervention; REDUCE-IT, Reduction of Cardiovascular Events with Icosapent Ethyl-Intervention Trial; STRENGTH, Long-Term Outcome Study to Assess STatin Residual Risk Reduction with EpaNova in HiGh Cardiovascular risk PatienTs with Hypertriglyceridemia; VITAL Rhythm Study, VITamin D and OmegA-3 TriaL Rhythm Study.

**Supplemental Figure 8** Dose-response association of EPA with new-onset atrial fibrillation.


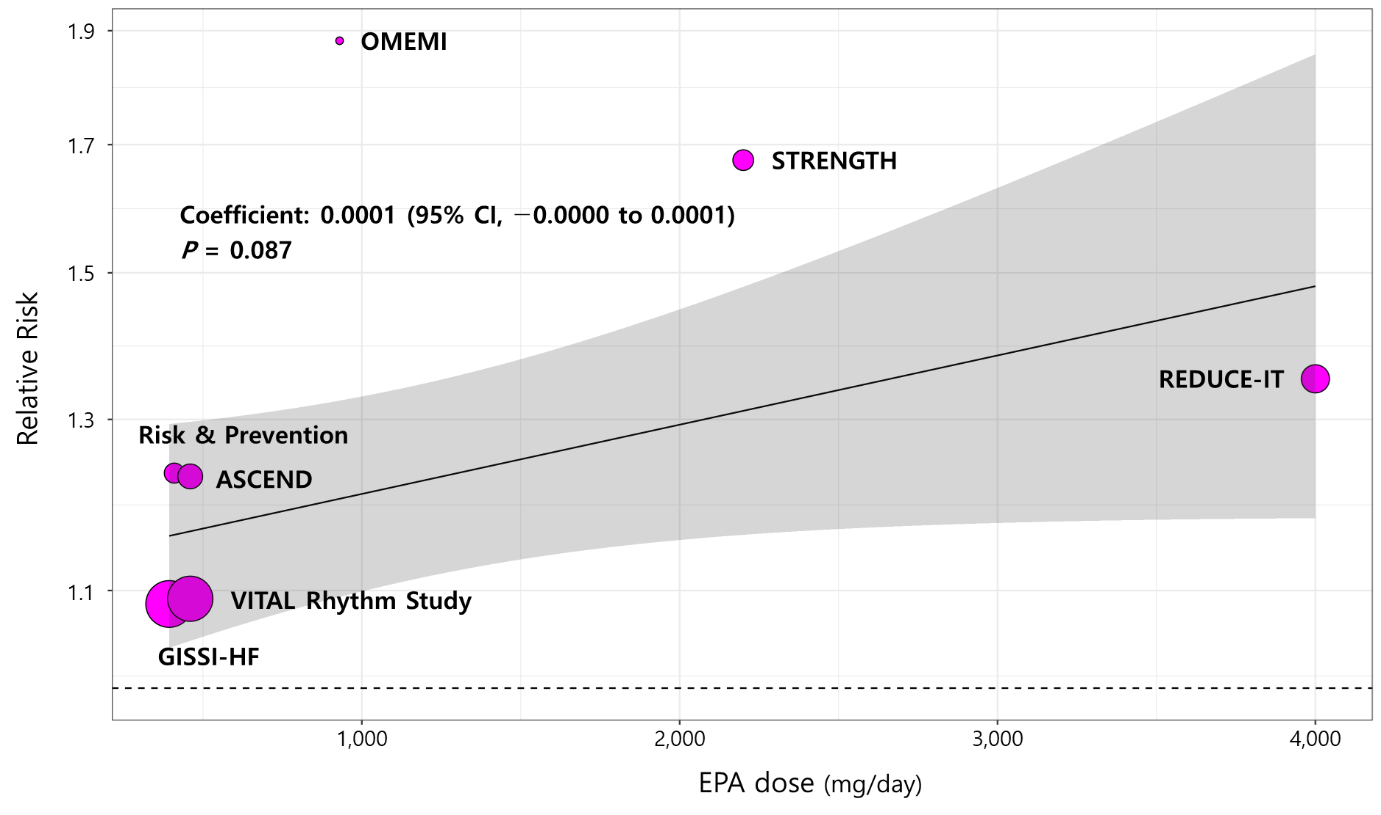


ASCEND, A Study of Cardiovascular Events iN Diabetes;CI, confidence interval; GISSI-HF, Gruppo Italiano per lo Studio della Sopravvivenza nell’Infarto miocardico-Heart Failure; OMEMI, OMega-3 fatty acids in Elderly patients with Myocardial Infarction; REDUCE-IT, Reduction of Cardiovascular Events with Icosapent Ethyl-Intervention Trial; STRENGTH, Long-Term Outcome Study to Assess STatin Residual Risk Reduction with EpaNova in HiGh Cardiovascular risk PatienTs with Hypertriglyceridemia; VITAL Rhythm Study, VITamin D and OmegA-3 TriaL Rhythm Study.
